# Constructing Ion‐Bridging Structure with Controlled Cracks in Plasticized PVC with Graphene for Highly Sensitive Strain Sensor with a Wide Strain Range

**DOI:** 10.1002/advs.202415998

**Published:** 2025-04-26

**Authors:** Hyosik Park, Mingyu Kim, Gerald Selasie Gbadam, Cheoljae Lee, Hyeonseo Joo, Sujeong Gwak, Bo‐Yeon Lee, Kyeong Nam Kim, Ju‐Hyuck Lee

**Affiliations:** ^1^ Department of Energy Science and Engineering Daegu Gyeongbuk Institute of Science and Technology (DGIST) Daegu 42988 Republic of Korea; ^2^ Department of Bionic Machinery Korea Institute of Machinery & Materials (KIMM) Daejeon 34103 Republic of Korea; ^3^ Division of Energy and Environmental Technology Daegu Gyeongbuk Institute of Science and Technology (DGIST) Daegu 42988 Republic of Korea; ^4^ Graduate School of Energy Science and Technology Chungnam National University Daejeon 34134 Republic of Korea; ^5^ Energy Science and Engineering Research Center Daegu Gyeongbuk Institute of Science and Technology (DGIST) Daegu 42988 Republic of Korea

**Keywords:** crack modulation, graphene, ion‐bridging, plasticized PVC gel, strain sensors

## Abstract

The gauge factor (GF) is a critical parameter for strain sensors, but it faces limitations in achieving high GF values across a wide strain range. This work proposes a novel approach to enhance resistance changes within strains through synergistically combining controlled‐crack sizing and an ion‐bridging structure. This ion‐conductive bridge forms at the interface between graphene and polyvinyl chloride (PVC) gel. Precise management of the crack initiation and propagation on graphene is achieved by controlling adhesion force between graphene and PVC gel. The resulting PVC gel/graphene‐based strain sensor featuring this synergistic design exhibits exceptional sensitivity. It achieves GFs of 635 (ε < 40%), 1.5 × 10^6^ (40% < ε < 80%), and 7.8 × 10^5^ (80% < ε < 100%) over a 100% stretching range. This innovative ion‐bridging construction enables precise control over bridge connectivity at the interface, mitigating graphene's inherent stretchability limitations and enhancing the GF of PVC gel, thereby enhancing strain sensor performance. The sensor detects bending motions and monitors angles within higher strain ranges, making it suitable for wearable applications in human motion tracking. Furthermore, a PVC‐based posture correction system distinguishes various motions, including shoulder band stretching, armband stretching, and even full squats, showcasing its practicality and versatility.

## Graphical abstract

A highly stretchable strain sensor is presented, that leverages controlled crack formation and ion bridging in a polyvinyl chloride gel/graphene to achieve exceptional sensitivity (gauge factor up to 1.5 × 10⁶) and mechanical stability. By optimizing plasticizer properties, the conductive pathways are finely tuned, enabling robust, real‐time monitoring of human motion for wearable electronics.

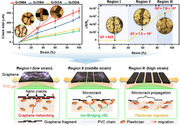

## 1 Introduction

In recent years, there has been a remarkable surge of interest in the advancement of sophisticated electronic skin (E‐skin) technologies.^[^ ^1^, ^2^ ^]^ With the ability to perceive various stimuli such as temperature variations, tactile sensations, and pressure, E‐skin is rapidly establishing its presence in diverse applications, including wearable electronics,^[^ ^3^ ^]^ human‐robot interfaces,^[^ ^4^ ^]^ and even posture correction systems.^[^ ^5^ ^]^ Strain sensors are essential to the effectiveness of E‐skin, playing a central role in capturing and monitoring bodily movements and changes. These sensors demand materials with specific characteristics: heightened sensitivity, stretchability, optimal Young's modulus for deformation, and unwavering stability. Notably, a resistive‐type sensor has been engineered to improve sensitivity, unlike a capacitive‐type sensor, which generates relatively lower sensitivity levels.^[^ ^6^ ^]^ However, achieving both ultra‐sensitivity (gauge factor, GF) and exceptional stretchability remains a significant challenge, especially for applications involving complex, large‐scale deformations such as joint movements and skin bending from attached strain sensors.^[^ ^7^, ^8^, ^9^ ^]^ Recent studies have emphasized the growing demand for strain sensors that maintain high GF performance across a broad strain range (<100%) and angular deformations (<180°), essential for real‐time monitoring in wearable devices.^[^ ^10^, ^11^ ^]^ For instance, wearable posture correction systems require strain sensors that can detect subtle changes in body position with high accuracy, necessitating both high sensitivity and mechanical robustness under repeated strain cycles. Therefore, the development of high‐GF strain sensors capable of sustaining reliable performance under relatively high strain conditions (≈100%) is crucial for advancing the next generation of flexible and wearable electronic applications.

Various stretchable resistive strain sensors with higher GFs have been reported via the development of materials and structural design strategies. As an approach regarding materials, incorporating various nanofillers^[^ ^12^, ^13^, ^14^, ^15^, ^16^, ^17^, ^18^ ^]^ with different dimensions, such as flake, nanowire, nanosheet, even ionic liquid, can be used to achieve high GF in strain sensors. However, most of these sensors still show low sensitivity and mechanical properties such as stretchability and stability. Moreover, specific structural strategies, encompassing mesh, wrinkles, cracks, and porosity, have been utilized to mitigate the limitations associated with the enhancement of sensing performance and stretchability. Nevertheless, it is noteworthy that their operation is confined to narrow strain ranges.^[^ ^19^, ^20^, ^21^, ^22^, ^23^, ^24^, ^25^, ^26^, ^27^, ^28^ ^]^ For instance, a two‐order system based on polydimethylsiloxane (PDMS) and 2D carbon materials developed by Ma et al.,^[^ ^20^ ^]^ exhibits a higher GF (88443) within a wide range of strains (350%). However, this system only attains a low GF of 18.5 at lower strains (≈80%), rendering it unsuitable for monitoring human motions within the strain range of 0% to 100%. Similarly, the combination of various conductive fillers and carbon paste in stretchable elastomer exhibited an ultra‐high GF of 1.8 × 10^6^, though constrained to a very narrow strain range (30–40%).^[^ ^19^ ^]^ This issue arises from the challenge of effectively modulating the internal resistance of active material under applied strain. Consequently, there is an urgent need to develop methodologies capable of effectively controlling internal resistance within a defined operational strain range. In other words, it is essential to design a crack‐modulated system that enables conductive materials to exhibit excellent conductivity (≈Ω) at low strain conditions while maintaining semiconductor characteristic (≈MΩ) even at high strain levels (> 50%). This can be achieved by facilitating the free movement of conductive elements within the system, allowing them to form dynamic bridges between modulated and disconnected conductors.

In this study, we present a meticulously designed approach leveraging the synergistic effects of constructing ion‐bridging and precise control of internal micro‐sized cracks to achieve an exceptionally high GF for a highly stretchable, wearable, and stable‐performing strain sensor. The polyvinyl chloride (PVC) gel is enriched with adipate plasticizer, facilitating facile movement within the PVC matrix, thereby serving as a semi‐conductive component that synergistically interacts with the highly conductive graphene film in the strain sensor. Notably, the control of crack size in the graphene plays a pivotal role in this system, modulating the adhesion between PVC branches and graphene units. The establishment of an ionic gel‐bridge between graphene layers ensures stable conduction pathways, consequently enhancing the operational strain range of the sensor. Additionally, optimized micro‐cracks in the graphene contribute to improvements in resistance changes in the strain sensor. Specifically, the PVC gel/graphene‐based strain sensor demonstrates a remarkable GF of 635 (ε < 40%), 1.5 × 10^6^ (40% < ε < 80%), and 7.8 × 10^5^ (80% < ε < 100%) over a wide stretching range, facilitating precise monitoring of human motion. This impressive GF achieved beyond the strain is attributed to the synergistic coupling of microcracks and ion‐bridging, resulting in the transition of conductive medium from graphene to PVC gel. This effectively mitigates the inherent limitations of graphene's lack of stretchability and the PVC gel's low GF. Moreover, the sensor exhibits superior performance in detecting real bending motions of human fingers, elbows, and knees, while accurately monitoring angles within higher strain ranges. Furthermore, we propose a novel posture correction system for band workouts, integrating a PVC gel/graphene‐based sensor onto a stretchable resistance band. This system effectively corrects various motions, including shoulder‐arm stretching motions and full squats, demonstrating the versatility and practical applicability of the developed sensor technology.

## 2 Results and Discussion

**Figure 1:**
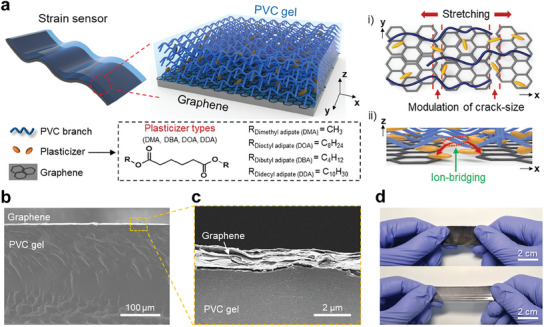
a) Schematic representation of PVC gel integrated with graphene featuring modulation of crack‐size and the formation of ion‐bridging at its interface. These i) modulation of crack‐size and ii) the ion‐bridging lead to large variation in resistance under stretching phase due to the coupling effect both decelerated ion migration and disconnection of ion‐bridge. b) Cross‐sectional scanning electron microscopy (SEM) image. c) Magnified view of the cross‐sectional SEM image illustrating the structure of PVC gel with graphene. d) Photographic images of PVC gel with graphene in its normal (top) and stretched (bottom) states.

**Figure** **1**a illustrates the strain sensor integrating a PVC gel with a graphene film, emphasizing the modulation of microcracks and the construction of ion bridging. Our methodology focuses on utilizing thermoplastic PVC as the primary matrix due to its robustness, durability, and chemical stability, making it well‐suited for various applications. Several environmentally friendly plasticizers, namely dimethyl adipate (DMA), dibutyl adipate (DBA), dioctyl adipate (DOA), and diisodecyl adipate (DDA), are introduced and uniformly dispersed within the PVC matrix through a high shear mixing method, employing a mixture dissolved in tetrahydrofuran (THF) (Figure S1, Supporting Information). These adipate plasticizers adopt a linear configuration with two carbonyl groups (C = O) and alkyl branches on both sides, creating a stretchable and ionic conductive PVC‐based gel. Crucially, a thin graphene film is carefully positioned beneath the PVC gel through a bar‐coating process (Figure S2, Supporting Information). As depicted in the inset of Figure S2 (Supporting Information), a multitude of PVC branches effectively interact with plasticizers and the graphene film. Two significant interactions primarily drive this structural arrangement: i) hydrogen bonds between the polar segment (H‐C‐Cl) of PVC branches and carbonyl groups (C = O) in plasticizers^[^ ^29^ ^]^ and ii) hydrogen bonds between the PVC chains and graphene defects (OH), as well as between plasticizers and graphene defects.^[^ ^30^, ^31^, ^32^, ^33^ ^]^ Based on the modulation of these interactions, which can be intuitively associated with adhesion between the PVC layer and the graphene layer, we can easily control the crack sizes of graphene during stretching phase. Above all, the combination of PVC gel and graphene forms ion‐bridging at the interface, facilitating effective electron transfer. The overall geometric structure of the PVC gel is measured using scanning electron microscopy (SEM), based on the uniform adherence of the graphene film (1200 nm) to the PVC gel, with a consistent thickness of 250 µm (Figure 1b,c). As depicted in Figure 1d, the stretchable PVC gel/graphene‐based strain sensor, presented in a deep black color, encompasses an effective area of 8 cm^2^.

**Figure 2:**
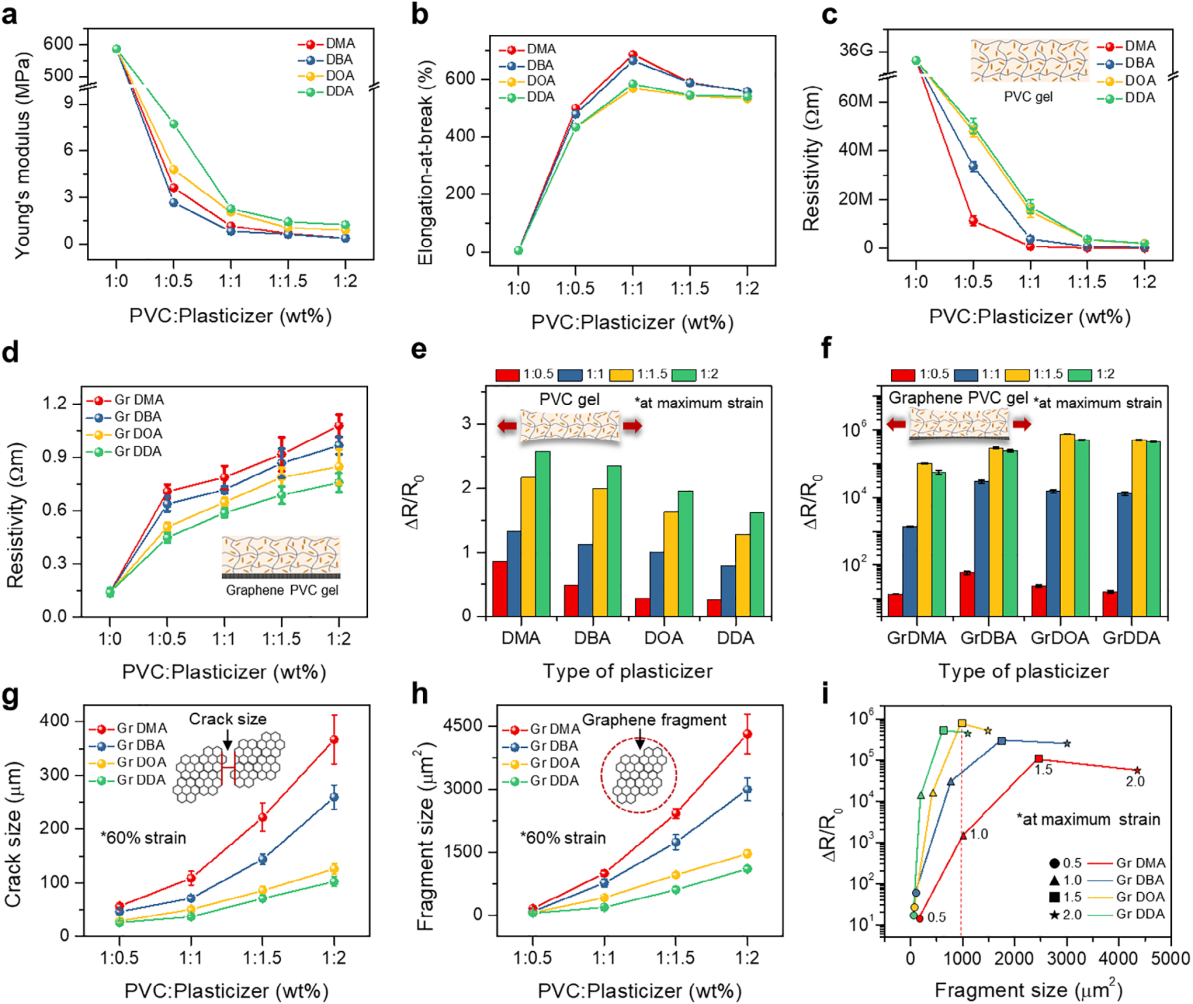
a) Young's modulus and b) elongation‐at‐break concerning various adipate plasticizers and their respective ratios. c,d) Resistivity profiles of PVC gel (c) and PVC gel integrated with graphene (d), based on different plasticizers and respective ratios. e,f) Resistance change profiles for the PVC gel alone (e) the PVC gel/graphene (f) with different plasticizers at varying mixing ratios under maximum strain limits. g,h) Crack size between adjacent fragments of graphene (g) and fragment sizes of graphene (h) with different ratios of various plasticizers. i) Resistance changes at maximum strain as a function of fragment size of graphene with different ratios and sizes of plasticizers.

For the purpose of designing an effective ionic conductive bridge, **Figure** **2** demonstrates the mechanical and electrical characteristics of PVC gels with graphene, incorporating four distinct plasticizers (DMA, DBA, DOA, and DDA). The plasticizers differ based on the length of their alkyl chains, which influences the mechanical characteristics and internal resistance of the PVC gel. We systematically characterized the Young's modulus, elongation‐at‐break, and ultimate tensile stress for PVC gels featuring varying plasticizers and mixing ratios (1:0, 1:0.5, 1:1, 1:1.5, 1:2), shown in Figure 2a,b and Figure S3 (Supporting Information). Based on the tensile stress‐strain curves (Figure S3, Supporting Information), all PVC gels manifest elastic behavior within wide ranges of strains, extending up to 400–700%, whereas the pristine PVC extends only up to 9.8%. As the plasticizer molecules become larger, the elastic modulus of the PVC gel increases. However, the elastic modulus decreases to ≈1 MPa at higher plasticizer concentrations. This value falls within the optimal elastic modulus range (kPa–MPa), ensuring sufficient flexibility and mechanical stability for direct wearable applications. This balance enables effective strain transmission, which is crucial for accurate human motion detection in real‐time monitoring.^[^ ^34^, ^35^, ^36^ ^]^ Also, a lower concentration of plasticizers results in the formation of a robust structure characterized by enhanced strength. Accordingly, the PVC gel incorporating DDA with a mixing ratio of 0.5 attains a peak ultimate tensile stress of ≈35 MPa (Figure S3f, Supporting Information). As the concentration of the plasticizer increases, there is a noticeable reduction in the ultimate tensile stress exhibited by the PVC gels. Notably, DDA, which has a longer alkyl chain compared to other plasticizers, exhibits a remarkable ultimate tensile stress. Typically, it tends to be that as the length of chain diminishes, a corresponding decrease in ultimate tensile stress ensues. The trend can be attributed to the longer alkyl chains leading to a more densely packed arrangement of PVC branches.^[^ ^37^, ^38^ ^]^ On the contrary, DMA, being the smallest plasticizer among them, achieves a remarkable elongation at break, reaching ≈700%. As the length of the alkyl chain increases, the elongation‐at‐break for the PVC gel experiences a reduction. Irrespective of the plasticizer type, a lower concentration thereof results in diminished elongation characteristics. Conversely, with increasing plasticizer concentration, elongation initially improves and then begins to decline after reaching its zenith at the 1:1 ratio. Consequently, as the alkyl chain length increases, the chains become more entangled, leading to higher tensile stress. Additionally, introducing more plasticizers generally improves the overall stretchability of the PVC gel. Remarkably, the PVC gels exhibit a more conductive nature in contrast to pristine PVC, as evidenced in Figure 2c, which explores the electrical resistivity of PVC gels with various plasticizers and mixing ratios. The results distinctly indicate that all PVC gels, irrespective of the lengths of alkyl chains within the plasticizers, manifest a semi‐conductive characteristic, with high resistivity of less than 5 × 10^7^ Ω∙m regardless of exceptionally high resistance of the pristine PVC (3.6 × 10^10^ Ω∙m). Of particular significance is the substantial reduction in resistivity for the PVC gel, primarily attributed to the utilization of a small‐sized plasticizer, namely DMA. Small plasticizers, characterized by their free‐flowing properties, possess the inherent capability to seamlessly permeate the structured branches of the PVC matrix, resulting in the formation of a semi‐conductive gel (1 × 10^7^ Ω∙m at the mixing ratio of 0.5). Moreover, a consistent trend emerges, demonstrating a progressive decrease in resistivity of 49,000 Ω∙m exhibited by DMA at a 1:2 mixing ratio with an increase in the concentration of plasticizer. This can be attributed to the abundance of plasticizer, which imparts conductive properties to the gel system. In addition, we further measured the Fourier‐transform infrared spectroscopy (FT‐IR) in various PVC gels, including different plasticizers, as shown in Figure S4 and Note S1 (Supporting Information). The introduction of a graphene film beneath the pristine PVC and PVC gels leads to a remarkable decrease in electrical resistivity, reaching levels as low as 0.1 Ω∙m as depicted in Figure 2d. Interestingly, the resistivity behavior exhibited by the pristine PVC and PVC gel with the graphene film contrasts entirely with that of the pristine PVC and PVC gel without the graphene film. Specifically, the smallest plasticizer, DMA, attains highest observed resistivity value of 1.1 Ω∙m at a plasticizer ratio of 2.0, regardless of the low resistivity of DMA alone. This phenomenon demonstrates that differences in adhesion between the PVC gel and graphene affect the size of fragments and cracks under peeling‐off strain, thereby altering the resistivity of the graphene PVC gel. Within the PVC gel, plasticizers not only increase the distance between PVC chains and reduce the number of PVC molecules per unit area, but also interact with graphene instead of PVC, disrupting the PVC–graphene bonding (Figure S5, Supporting Information).^[^ ^39^ ^]^ When the adhesion increases, the interaction between graphene and PVC is strengthened, leading to smaller graphene fragments and reduced crack sizes, which in turn enhances electron tunneling and lowers the resistivity. As shown in Figure 2e, we present the strain sensing characteristics of the PVC gel with different plasticizers and concentrations under strains of up to their maximum strain limits (0.5 ratio: 60%, 1.0 ratio: 80%, 1.5 and 2.0 ratios: 100%). Also, we included a supportive description and characterization in Figures S6–S9 (Note S2, Supporting Information) Furthermore, achieving high resistance change (ΔR/R_0_) in the PVC gel with graphene requires systematic optimization of both the concentration and chain length of the plasticizer, as illustrated in Figure 2f and Figures S10–S13 (Supporting Information). Increasing the plasticizer concentration generally leads to an increase in ΔR/R_0_. Additionally, the alkyl chain length significantly influences ΔR/R_0_, with longer chains enhancing its value. The maximum ΔR/R_0_ achieved is ≈10^6^ for DOA at a 1:1.5 ratio, primarily due to the modulation of crack size. We performed a systematic statistical analysis of fragment size at varying plasticizer concentrations as shown in Figure 2g,h and detailed in Tables S1 and S2 (Supporting Information), under 60% strain. Under these conditions, the maximum crack size between adjacent fragments is ≈350 µm, while the fragment size can be precisely controlled by adjusting the concentration and size of plasticizer, reaching ≈4500 µm^2^ with DMA at a 1:2 ratio. In addition to crack and fragment size, we quantified the crack number density to further explain the effect of plasticizer properties on crack formation and to depict the correlation among crack number density, crack size, and fragment size, as shown in Figure S14 and Table S3 (Supporting Information). Increasing the plasticizer concentration leads to a decrease in crack number density, and smaller plasticizer molecules yield an even lower density. These measurements help explain how plasticizer properties influence crack formation, and a strong correlation with both crack size and fragment size is observed. Consequently, the most effective plasticizer in this system, yielding a ΔR/R_0_ of ≈10^6^ is DOA a 1:1.5 ratio, which achieves an optimal graphene fragment size of ≈1000 µm2 at maximum strain (Figure 2i).

**Figure 3:**
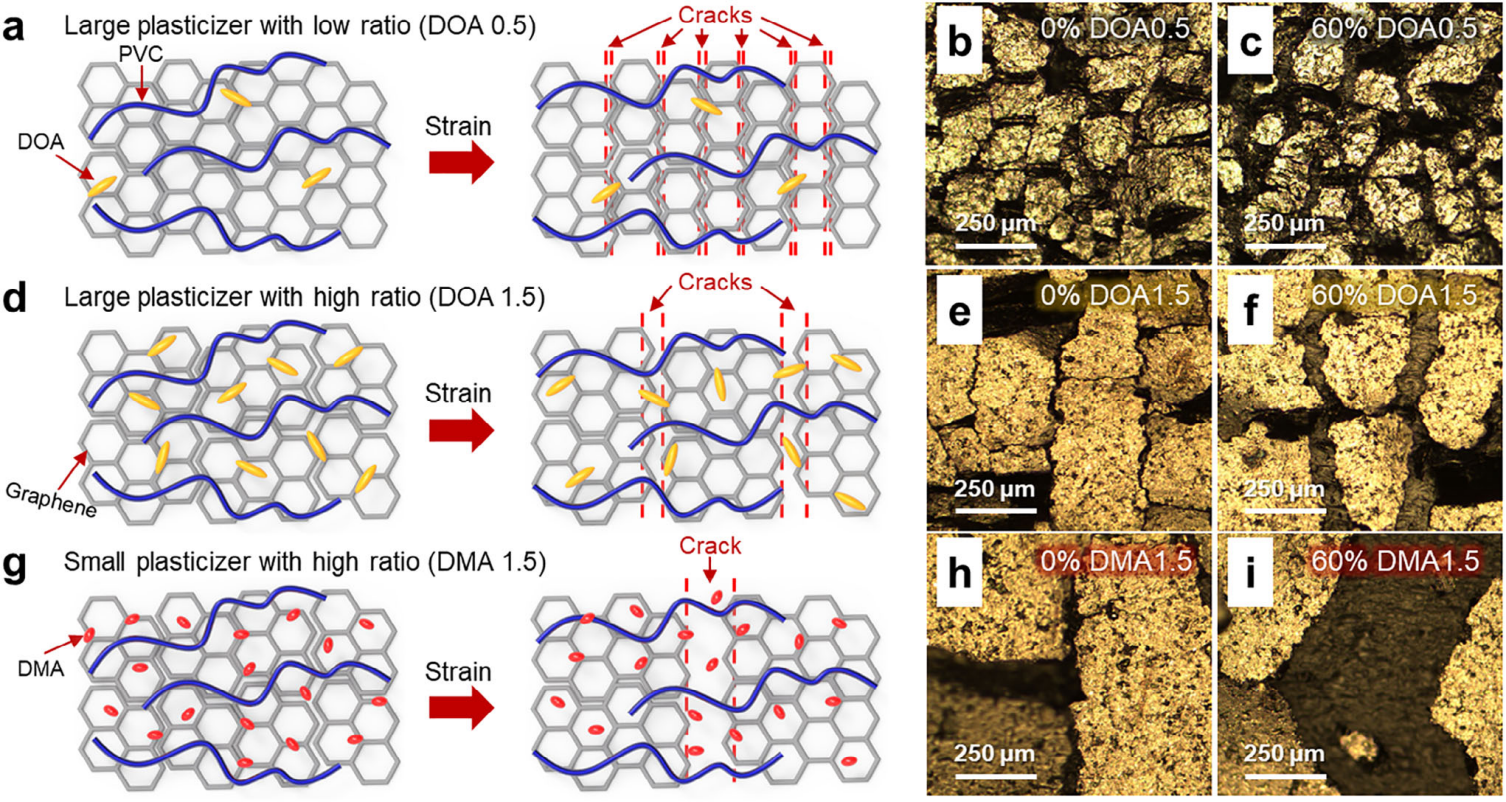
The correlation between plasticizer concentration and size with crack size. Schematic mechanism (a,d,g) and optical microscopy (OM) images each at 0% (b,e,h) and 60% strain (c,f,i) of three model variants: a–c) large plasticizer with low ratio (DOA, 1:0.5), d–f) large plasticizer with high ratio (DOA, 1:1.5), and g–i) small plasticizer with high ratio (DMA, 1:1.5).

As previously noted, the concentration and size of plasticizers significantly influence the microcrack size within graphene layers by modulating the adhesion between graphene and PVC gel. The mechanism of crack size modulation is illustrated in **Figure** **3**. Based on Figure S5 (Supporting Information), large plasticizers at low concentrations induce strong interactions between graphene and PVC gel. Under strain, this strong adhesion between PVC and graphene results in numerous fine cracks due to relatively weak interlayer connectivity within the graphene (Figure 3a–c). However, at higher plasticizer concentrations, a larger amount of plasticizer interacts with PVC, weakening the adhesion between graphene and PVC. When strain is applied, this weakened interaction at the interface leads to slip phenomenon, strengthening the connectivity among graphene layers. Although some regions still exhibit strong PVC‐graphene adhesion, cracks originate in these areas, and the cracks propagate outward, leading to larger fragments and crack sizes compared to the previous condition (Figure 3d–f). Finally, when using the smallest plasticizer, more plasticizer molecules bind tightly with PVC, resulting in extremely weak PVC‐graphene adhesion. Consequently, significantly larger fragments and cracks are formed under strain (Figure 3g–i). We also conducted a comparison of graphene crack sizes under different strains using an optical microscope (OM), as depicted in Figures S15–S19 (Supporting Information). As strain increases in both directions, the size of cracks in the graphene also increases. The crack size increases with the concentration of plasticizers. Upon applying strain, large plasticizers (DOA, DDA) with a minimal quantity result in the formation of numerous yet fine cracks on graphene layers. We also employed atomic force microscopy (AFM) to image crack modulation at different plasticizer concentrations, as shown in Figure S20 (Supporting Information). The DOA at 0.5 exhibited a rough graphene surface due to smaller fragments, while the DOA at 1.5 displayed a relatively smooth graphene surface because of larger fragments.

In addition, we developed a theoretical model to clarify how plasticizer properties affect crack formation. We propose that the binding energy (E) at the PVC gel–graphene interface can be expressed as below,

(1) $$E\propto n_{PVC}/n_{Plasticizer}\propto 1/R$$

where *n_PVC_* and *n_Plasticizer_* represent the number of moles of PVC and plasticizer in PVC gel, respectively, and R denotes the resistivity of the PVC gel with graphene. In this model, a higher PVC/Plasticizer molar ratio (*n_PVC_*/*n_Plasticizer_*)—indicating lower plasticizer concentrations or larger plasticizers—leads to an increase in binding energy, resulting in smaller graphene fragments and crack sizes, enhancing electron tunneling, and lowering resistivity. We further relate the crack and fragment sizes to the binding energy and resistivity as follows:

(2) $$S_{Crack}\propto S_{Fragment}\propto \frac{1}{E}\propto \frac{n_{Plasticizer}}{n_{PVC}}\propto R$$

where *S_Crack_* and *S_Fragment_* are the sizes of crack and fragment, respectively. To validate this theoretical model, we confirmed the relationship between the PVC/Plasticizer molar ratio and crack size as shown in Figure S21 (Supporting Information). As the PVC/Plasticizer molar ratio increases, crack size decreases, which follows an inverse correlation. According to this theoretical model, when the molar ratio is below 4 × 10⁻⁴, the crack size exceeds 200 µm; between 4 × 10⁻⁴ and 8 × 10⁻⁴, the crack size ranges from 100 to 200 µm; between 8 × 10⁻⁴ and 1.5 × 10⁻^3^, the crack size is between 50 and 100 µm; and for molar ratios above 1.5 × 10⁻^3^, the crack size is less than 50 µm. Thus, we can effectively control graphene's crack and fragment sizes by adjusting the type and concentration of plasticizer—key factors determining the PVC/plasticizer molar ratio.

**Figure 4:**
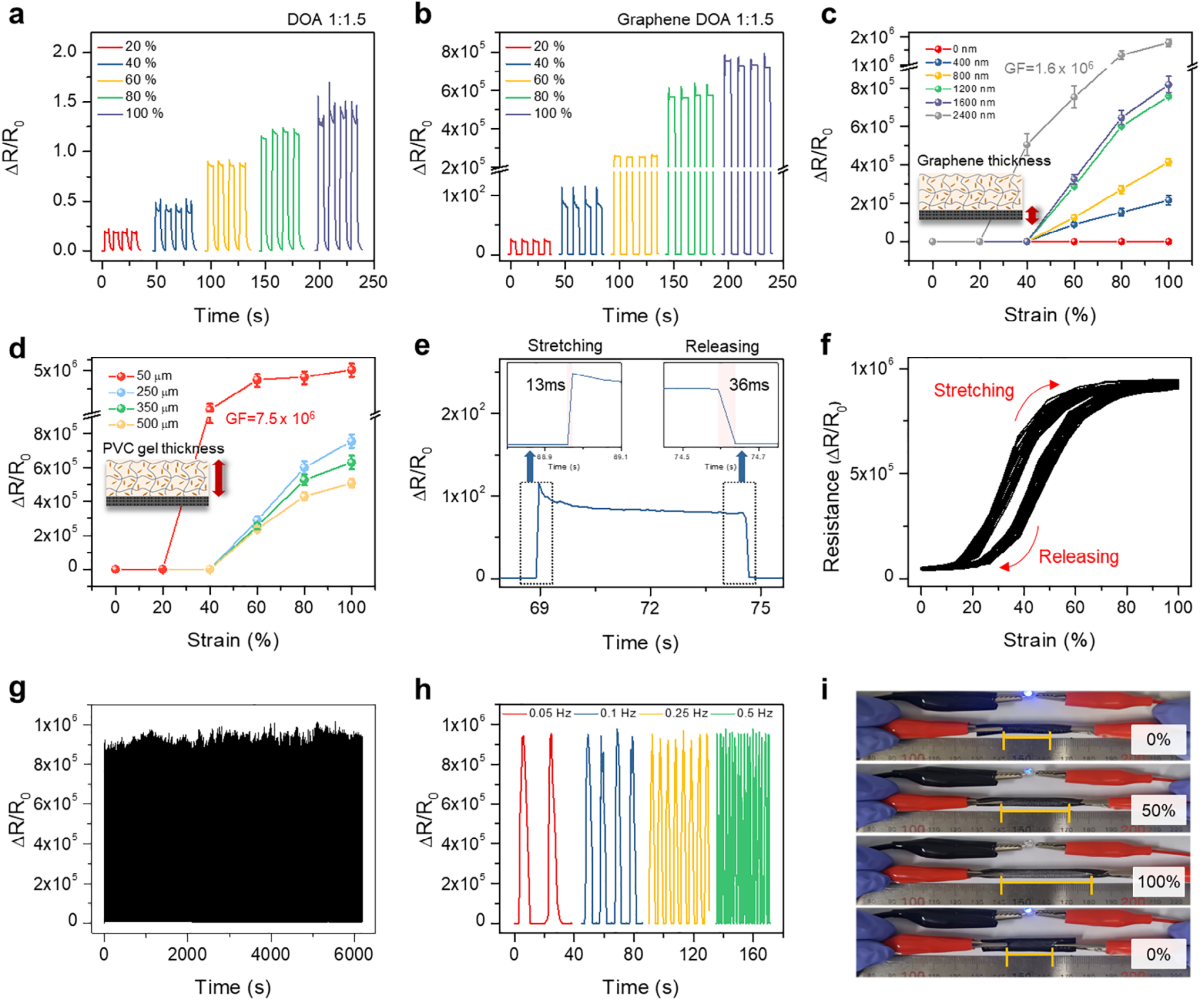
a,b) Resistance changes of the PVC gel‐based sensor (DOA, 1:1.5) without graphene (a) and PVC gel/graphene‐based sensor (DOA, 1:1.5) (b) in reaction to different stretching strains (20–100%). c) Resistance changes corresponding to different thicknesses of the graphene film. d) Profile in resistance change in relation to the thickness of PVC gel. e) Response (13 ms) and recovery (36 ms) time of the sensor during stretching‐releasing process. f) Hysteresis loops of the sensor response after 40 cycles of repeated stretching and releasing up to 100% strain. g) Stability assessment of the sensor over an extended operational life of 1500 cycles, with an inset graph providing a magnified view of stable electrical signals. h) Resistance changes across a range of frequencies, from 0.05 to 0.5 Hz. i) Photographic evidence displaying illuminated LEDs to illustrate the conductive properties of the PVC gel/graphene during stretching and releasing motions.

As shown in **Figure** **4**, we present the strain sensing characteristics of our sensor under strains of up to 100%. Initially, we systematically measured ΔR/R_0_ of the optimized PVC gel (DOA, 1:1.5) and PVC gel with graphene under strain over the same period, as shown in Figure 4a,b. Similar to traditional resistive sensors, the ΔR/R_0_ (≈1.5 at 100%) of PVC gel alone demonstrates stable performance and increases with strain, attributed to the rise in internal resistance under deformation (Figure 4a). Incorporating graphene beneath the PVC gel significantly improves ΔR/R_0_, reaching ≈8 × 10^5^ with similarly stable performance (Figure 4b). To determine the optimal thicknesses of PVC gel and graphene for the strain sensor, we conducted a comprehensive assessment of ΔR/R_0_ at varying strains, as detailed in Figure 4c,d. With PVC gel thickness maintained at 250 µm, we varied the graphene thickness to 400, 800, 1200, 1600, and 2400 nm. ΔR/R_0_ increased with thicker graphene layers, with 1200 nm graphene achieving a significantly heightened GF of 1.5 × 10^6^. This enhancement is primarily attributed to the lower initial resistance of the thickest graphene compared to graphene with a thinner thickness. However, increasing the thickness to 2400 nm graphene raised the GF to 2.5 × 10⁶. Notably, the 2400 nm graphene exhibited an earlier onset of Region II at 20% strain, likely because the increased thickness enhances graphene–graphene interactions, thereby reducing the PVC gel–graphene binding energy and resulting in larger fragment sizes. Despite the higher GF at 2400 nm graphene, it showed significantly reduced mechanical stability under repeated strain cycles due to increased internal stress and weaker interfacial adhesion (Figure S22, Supporting Information). Thus, while increasing graphene thickness improves GF by reducing R₀, it also compromises mechanical durability. Based on our results, a graphene thickness of 1200 nm provides the optimal balance between sensitivity and structural reliability for our device. Ultimately, graphene with a thickness of 1200 nm was selected as the optimal material for the final device. Conversely, with a fixed graphene thickness of 1200 nm and varied PVC gel thicknesses at 50, 250, 350, and 500 µm, ΔR/R_0_ decreased as the thickness of PVC gel increased. This phenomenon can be attributed to the fact that a thicker gel results in lower resistance. The sensor with 50 µm PVC gel exhibited a dramatic resistance change, achieving a maximum GF of 7.5 × 10^6^ within the strain range of 20–40%. This value represents the highest GF reported in stretchable strain sensors to date. However, it is important to note that the operational strain range (20–40%) displaying this maximized value is relatively narrow. Also, a shoulder peak is observed beyond a strain of 60%, indicating irreversible deformation across the gel. Variations in the thickness of PVC gel and graphene lead to distinct effects on adhesion behavior and electrical properties, ultimately determining the sensor's reliability. Considering these findings, we selected PVC gels with a thickness of 250 µm and graphene with a thickness of 1200 nm as the final sensor configuration. The sensor demonstrated rapid response and recovery times in the millisecond ranges (13 and 36 ms, respectively) in Figure 4e, highlighting its capability for high‐speed signal acquisition in dynamic motion monitoring. Figure 4f presents the sensor response curves following 40 cycles of repeated stretching and releasing strains up to 100%, demonstrating hysteresis behavior. This phenomenon is commonly observed in other studies involving resistive sensors and is attributed to a delay in the rearrangement of conductive pathways under strain. While hysteresis can influence sensing accuracy, the observed behavior remains within an acceptable range for practical applications. Furthermore, the sensor exhibited impressive stability over 1500 cycles (at a frequency of 0.25 Hz) under 100% strain (Figure 4g). No observable resistance drops occurred in measured signals throughout the entire operation, confirming the sensor's long‐term reliability for practical application. Additionally, this sensor successfully operated under various frequencies, ranging from 0.05 to 0.5 Hz as illustrated in Figure 4h, without any abnormal signal occurrences. These results collectively highlight the sensor's robustness, rapid responsiveness, and mechanical resilience, making it well‐suited for real‐time human motion detection and wearable electronics. As shown in Figure 4i, the sensor exhibited excellent conductivity and functioned as a conductive medium without strain. As the strain levels increased up to 100%, this conductive property diminished. However, the conductivity was successfully restored upon release, returning the sensor to its original state. Additionally, we fabricated 20 sensors to compare performance, analyze the error between sensor‐measured and actual strain, and conduct statistical analysis, as shown in Figure S23 and detailed in Table S4 (Supporting Information). Although slight performance variations were observed, the ΔR/R_0_ values remained consistent, demonstrating robustness despite the inherent randomness in the distribution of plasticizer and graphene (Figure S23b, Supporting Information). Based on these resistance changes, the corresponding strain values were back‐calculated (Figure S23c, Supporting Information) and compared with the applied strain levels, showing strong agreement.

**Figure 5:**
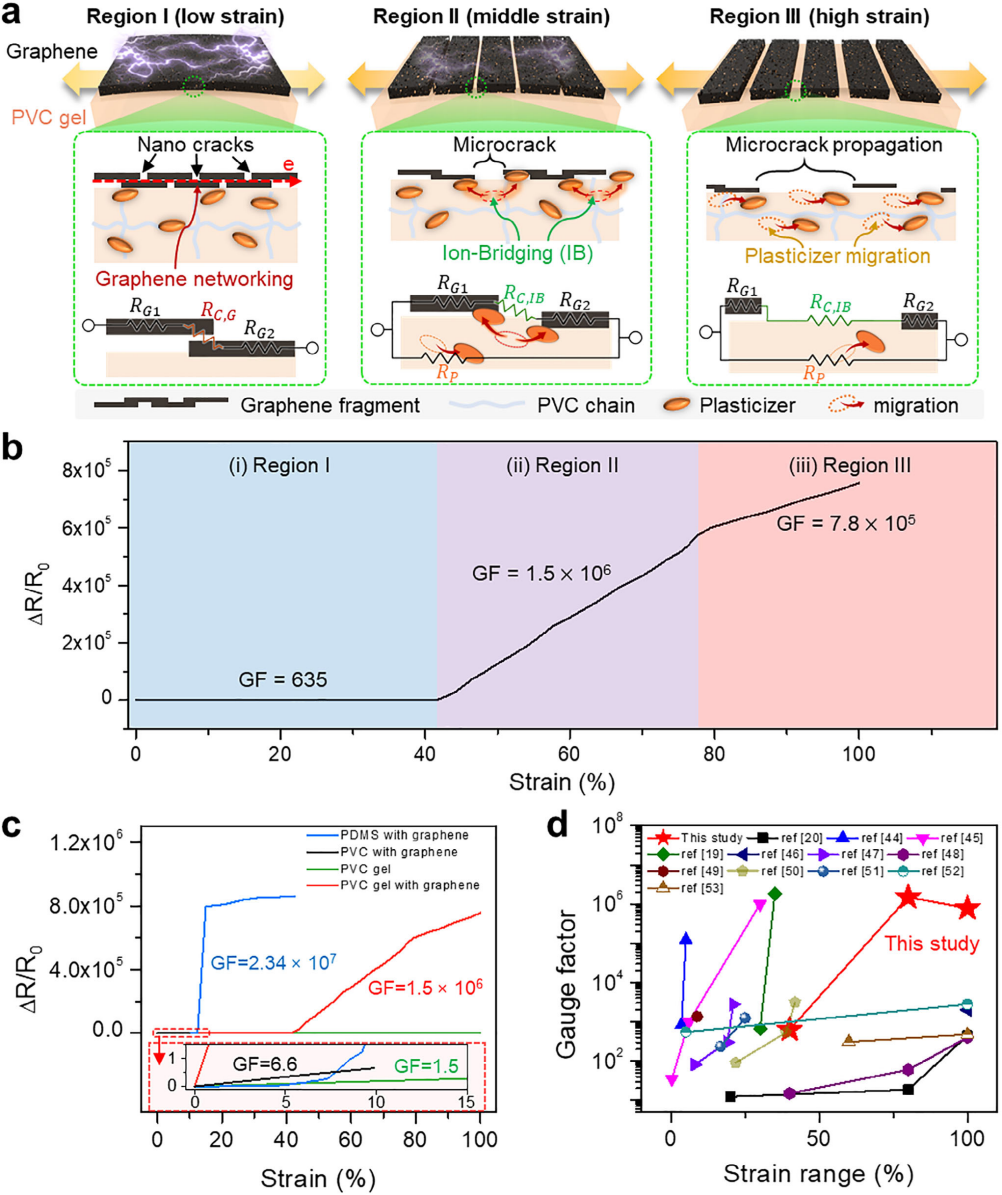
a) Operational mechanism of the PVC gel/graphene‐based strain sensor based on crack modulation and ion‐bridging, exhibiting three distinct behaviors. b) Resistance changes of PVC gel with graphene, depicting three regions of the strain sensor's behavior across varying strain levels. c) Profiles illustrating resistance changes for PDMS integrated with PVC with graphene (black line), graphene (blue line), and PVC gel integrated with graphene (red line), standard PVC gel (green line) under 100% strain conditions. d) Comparative analysis of gauge factors and operational strain ranges in relation to previous strain sensors.

To comprehend the underlying mechanism (**Figure** **5**a), we describe that the total resistance (*R_T_*) of the G‐PVC film can be expressed with parallel‐connected graphene and PVC gel as per Equation (3),^[^ ^13^, ^40^, ^41^ ^]^

(3) $$R_{T}=\left(R_{G}R_{C}+2R_{G}R_{P}+R_{P}R_{c}\right)/\left(R_{G}+2R_{c}+R_{P}\right)$$

where *R_G_*, *R_P_*, and *R_c_* denote the resistances of graphene, PVC gel and contact resistance at the interface, respectively as shown in Figure S24 (Supporting Information). According to this equation, the total resistance of this system converges toward the resistance value of the material with lower resistivity between the two substances. Thus, in order to exhibit a high GF of strain sensors, the system should be designed with an exceptionally low resistance without strain. Also, the adhesion at the interface should be good to maintain low contact resistance. Initially, in the absence of strains, the graphene film, and the PVC chains in PVC gel, maintain a connection with each other at interface, resulting in low initial resistance. Significantly, the use of large plasticizers, such as DOA or DDA with low concentration among the available options, amplifies the electrical conductivity of the G‐PVC gel film. As we discussed before, this enhancement is attributed to the improvement of adhesion between graphene layers, which effectively enhances electrical connectivity between graphene layers. Within Region I (0–40%, *R_G_* <*R_P_*) in Figure 5a,b‐(i), as the applied strain increases, numerous fine cracks form in the graphene film. This disconnection mechanism causes a reduction in electrical conductivity, as expressed by Equations (4)–(6).^[^ ^42^ ^]^

(4) $$R_{C,G}=a/S$$

In Equation (2), *S* denotes the overlapping area between graphene sheets, *a* is the constant value associated with surface condition, contact pressure, and electrical resistivity. The equation suggests that a larger overlapping area between graphene sheets and between graphene and plasticizer results in a decrease in the total resistance. The overlapping area diminishes when strain is applied, as indicated by Equation (5):

(5) $$S_{x}=S_{0}\left(1-b\varepsilon \right)$$

Here, *S_x_* represents the overlapping area under a strain of x, *S_0_* is the overlapping area at the strain of 0%, b is a constant related to the overlapping mode, and ε is the strain. Consequently, based on the following Equation (6), *ΔR/R_o_* decreases under applying the strain.

(6) $$\Delta R_{G,x}/R_{G,0}\approx ab\varepsilon /\left(S_{0}R_{0}\right)$$

Thus, in the case of graphene with PVC gel under the low strain, a significant portion of the graphene remained interconnected, providing an additional conductive pathway due to the existence of mobile plasticizers at the interface between graphene and PVC gel, extending this Region I based on this disconnection mechanism with a low GF of 635.

Region II (40–80%) showed a significant increase in ΔR/R_o_ as shown in Figure 5a,b‐(ii). This phenomenon can be primarily attributed to the rapid disconnection of ion‐bridge (*R_C,IB_*) at the interface and the appearance of microcracks in graphene. Within the corresponding strain range, a fundamental shift occurs in the major conductive mediator, transitioning from graphene to PVC gel as graphene becomes completely disconnected (*R_G_* >> *R_P_*). Surely, the resistance of PVC gel also increases with increasing strain due to geometrical changes, which can be expressed as a function of the strain ε.^[^ ^43^ ^]^

(7) $$\Delta R_{P,x}/R_{P,0}=\varepsilon^{2}+2\varepsilon$$

During this phase, graphene assumes a role as a supportive station, facilitating electrical conductivity, rather than serving as the primary conduction pathway in Region I. In essence, this means that a less ion‐conductive plasticizer can readily migrate to each of these supportive stations. However, when tensile strain is applied, the distance between these graphene stations increases, making it more challenging for the plasticizer to reach them. Furthermore, the mobility of plasticizer in stretched PVC matrix diminishes. Consequently, the substantial ΔR/R_o_ and exceptional GF of 1.5 × 10^6^ observed during this phase can be attributed to the synergistic effect of rapid loss of the ionic conductive bridge and the optimized fragment and crack sizes, which alter the major conductive mediator, as detailed in the Note S3 (Supporting Information). We also performed Raman analysis to verify the ion‐bridge effect as shown in Figure S25 (Supporting Information). In PVC gel containing DOA1.5, the CH_3_ symmetric stretching (2870 cm^−1^) and CH_2_ asymmetric stretching (2915 cm^−1^) peaks were strong due to CH_2_ and CH_3_ groups in both PVC and the plasticizer. However, upon introducing graphene, these peaks decreased significantly, indicating strong interactions between PVC and graphene and a corresponding restriction in plasticizer mobility. The observed ion‐bridge effect is likely promoted by hydrogen bonding between the plasticizer and graphene defects, with these defect sites acting as active centers for ionic interactions that effectively form bridges between polymer chains (Figure S26, Supporting Information).

Within Region III (80–100%) in Figure 5a,b‐iii), the PVC gels exhibited internal elastic deformation, characterized by geometrical changes, leading to a significant surge in resistance change, as explained by Equation (7). Nevertheless, it is noteworthy that the rate of ΔR/R_o_ gradually decelerated within this region (GF = 7.8 × 10^5^), suggesting the onset of a saturation effect and the existence of the propagated and microcracked graphene as conductive station.

The evidence supporting ion‐bridging can be categorized into three primary observations: i) Differences in resistance trends between PDMS with graphene and PVC gel with graphene, ii) Resistance differences between PVC gel and PVC gel with graphene under extreme strain, and iii) Comparisons with PVC neat with graphene.

i) Regarding the resistance changes in materials containing only graphene (e.g., PDMS with graphene), as shown in the revised Figure 5c, the resistance tends to increase significantly beyond a certain strain (12%), failing to maintain a low resistance. This is attributed to the absence of a mobile medium that acts as a bridge. Conversely, in PVC gel with graphene, even when graphene fragments are disrupted by strain, the plasticizers present in the PVC matrix effectively migrate between the graphene fragments, causing the resistance to increase gradually. This demonstrates that plasticizers can act as mediators, connecting graphene fragments. ii) Additionally, we compared the resistance of PVC gel and PVC gel with graphene under extreme strain (100% strain), where graphene is completely fractured (as shown in Figure S27, Supporting Information). The results indicate that even after complete graphene disconnection, the resistance of PVC gel with graphene (≈10 GΩ) is more than four times lower than that of PVC gel alone (≈40 GΩ). This is due to the plasticizers in the PVC gel, which migrate freely between graphene fragments, reducing resistance despite the fracture. iii) Finally, PVC neat with graphene exhibits a low GF (6.6) and limited strain tolerance (<10%). The addition of plasticizers significantly enhances both the GF and strain range, which can be attributed to the mobility of the plasticizers. This mobility facilitates the ion‐bridging effect, linking graphene fragments and improving performance. In summary, these results confirm that the plasticizers in PVC serve as mediators, establishing an ion‐bridging effect that connects graphene fragments. That's why our device, based on PVC gel with graphene, characterized by remarkable GF performance across a wide range of strains, was categorized into three distinct regions, as depicted in Figure 5b. To showcase an excellence of the PVC gel incorporating a graphene film, as depicted in Figure 1b, we conducted an experimental comparison of the resistance profiles of four representative sensors: i) PVC with graphene, ii) PDMS with graphene, iii) PVC gel with graphene, and iv) only PVC gel, as shown in Figure 5c. First, PVC alone cannot be much stretched (≈10%, refer to Figure S3a, Supporting Information) and generated a GF of 6.6, demonstrating a poor sensing performance. As anticipated, the graphene on PDMS exhibited a limited operational strain range of less than 15%, with an exceptionally high GF of ≈2.34 × 10^7^ within the narrow strain range of 12–15%. In contrast, when used in isolation, the PVC gel alone demonstrated remarkable stretchability, reaching up to 550%, but with a significantly low GF of 1.5. Thus, these two devices individually failed to meet the criteria for high GF performance under long‐range strains. In contrast, the PVC gel with graphene film exhibited a high GF of 1.5 × 10^6^ over a wide range of strains extending up to 100%. We compared our sensor's performance with other reported strain sensor devices (Figure 5d),^[^ ^19^, ^20^, ^44^, ^45^, ^46^, ^47^, ^48^, ^49^, ^50^, ^51^, ^52^, ^53^ ^]^ revealing that our PVC gel with graphene achieved the highest GF within a strain up to 100%.

**Figure 6:**
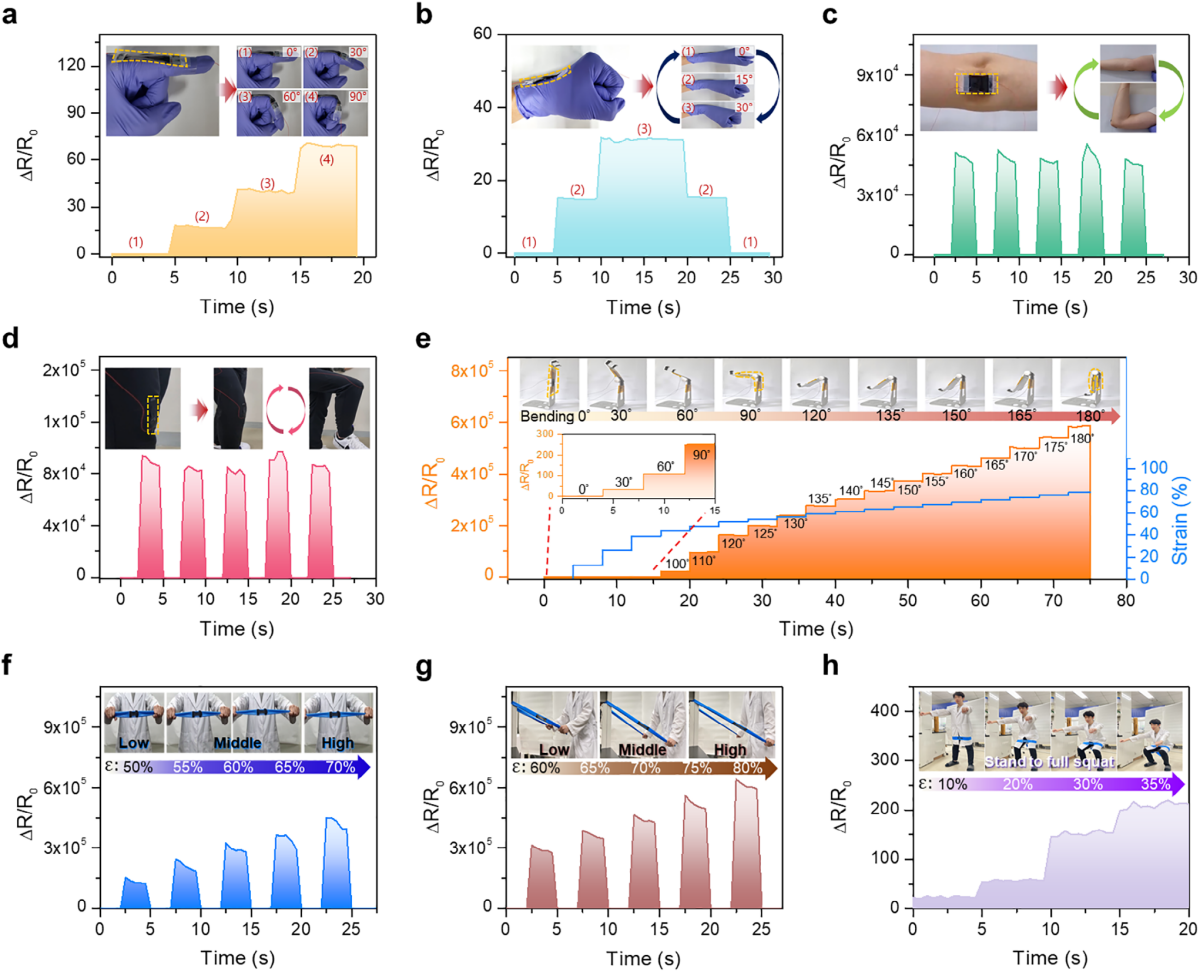
a) Resistance changes in response to different angles (0°, 30°, 60°, 90°) during finger bending motions. b) Resistance change with varied angles (0°, 15°, 30°) during wrist motions. c) Repeated resistance signals during arm bending motion at 90°. d) Repetitive electrical signals reflecting resistance changes during knee bending movement at 90°. e) Resistance change profile across a wide bending strain range (0°–180°) with the corresponding converted strains for a standing tablet. f,g) Resistance changes in strain sensor attached to a resistive band during shoulder band stretching (50–70%) f) and armband movements (60–80%) g). h) Resistance changes at various stages of squat motion (quarter, half, and full).

Finally, the PVC gel/graphene‐based sensor demonstrates real‐time monitoring of various human motions while worn on the body, as shown in **Figure** **6**. As shown in Figure 6a, the sensor successfully detects finger bending at angles of 30° (ΔR/R_0_ = 18), 60° (ΔR/R_0_ = 40), 90° (ΔR/R_0_ = 70). Figure 6b illustrates the sensor monitoring wrist bending, with resistance changes of ΔR/R_0_ = 15 at a 10° angle and ΔR/R_0_ = 31 at a 30° angle. Figure 6c,d show electrical signals corresponding to arm (at a 90° angle) and knee bending (at a 135° angle). The strain from arm and knee bending motions can be estimated from the resistance changes, ≈5 × 10^4^ and 9 × 10^4^, corresponding to strains of ≈45% and 48%, respectively. It is important to note that some inconsistency in signals may be caused by generating inaccurate human motions. Rather, it can be useful as a highly accurate sensor for posture correction in the intermediate range of strain. The sensor was also applied to monitor extreme deformations, such as tablet stand folding, as illustrated in Figure 6e. The tablet stand can be folded up to the angle of 180°, where the relative lower angles can be detected by using the sensor (ΔR/R_0_ = 35, 110, 250 at the angles of 30°, 60°, 90°) as shown in the inset of Figure 6e. The higher angles of the tablet stand can be accurately differentiated by using the sensor. At 100° and 180° angles, the corresponding resistance changes are recorded as 2.2 × 10^4^ and 5.8 × 10^5^, respectively. Additionally, the sensor accurately detected fine angular changes (≈5°) between 120° and 175°, with values (ΔR/R_0_ = 1.6 × 10^5^, 2.0 × 10^5^, 2.4 × 10^5^, 2.7 × 10^5^, 3.0 × 10^5^, 3.3 × 10^5^, 3.7 × 10^5^, 4.0 × 10^5^, 4.3 × 10^5^, 4.6 × 10^5^, 5 × 10^5^, 5.4 × 10^5^) exhibiting a significant increase in resistance change, as shown in Figure 6e. These angular changes were converted to strain using the equation ε = *h*/2*r*, where *ε* is the bending strain, *h* is the thickness of the sensor, and *r* is the radius of curvature. At higher strain levels (angles), the sensor effectively distinguishes subtle strains or angle differences, enabling precise monitoring for posture correction. In another application, the sensor was integrated into a resistive band for posture correction during shoulder workout, as shown in Figure 6f and Movie S1 (Supporting Information). The sensor successfully detected stretching motions of the shoulder band within strain ranges of 50–70%. When maintaining the correct posture, the sensor showed a remarkably stable electrical signal, particularly at high strain (ΔR/R_0_ = 4.5 × 10^5^ at the strain of 70%). However, deviations from the intended posture resulted in unstable resistance signals or decreased signal quality, as shown in Figure S28 (Supporting Information). Subsequently, we anchored the band to a fixed point and engaged both arms to perform controlled stretching exercise, as illustrated in Figure 6g and Movie S2 (Supporting Information). Our results revealed discernible variations in resistance levels, a testament to the ultra‐high sensitivity of the sensor. We categorized these exercises into three stages: low, middle, and high stretching, involving strain levels of 60%, 65%, 70%, 75%, and 80%, respectively. While some signals exhibited minor reductions, these variations are likely due to the inherent variability in human movements. Lastly, the strain sensor was used for posture correction during squat exercises, with the band wrapped around both thighs, as shown in Figure 6h and Movie S3, Supporting Information). In the initial phase of the workout, stretching of the band triggered a ΔR/R_0_ value of 30 (10% strain), allowing for real‐time monitoring of movement initiation. The squat routine was methodically divided into three stages (quarter, half, and full), and the sensor consistently detected resistance changes at each stage (ΔR/R_0_ = 50, 150, 200). This allowed for the precise assessment of the positioning and tension in the lower extremities, critical factors in the execution of squat motions. Additionally, Figure S29 (Supporting Information) provides evidence of the sensor's capacity for accurate motion detection even during repeated movements.

## 3 Conclusion

In summary, our study highlights the efficacy of employing PVC gels integrated with graphene films to engineer a highly sensitive strain sensor. This sensor has demonstrated remarkable effectiveness in monitoring human motions and object deformations within a wide strain range, reaching ≈100%. Particularly noteworthy is the strain sensor based on plasticized PVC gel, featuring a graphene thin film beneath the gel, which exhibited an outstanding GF of 1.5 × 10^6^ over the range of 40% < ε < 80%. Moreover, it showcased exceptional durability, enduring 1500 cycles of stretching motion. This achievement arises from the synergistic combination of crack size control and the establishment of an ion‐bridging structure. The modulation of crack size in the graphene film is achieved by controlling adhesion between PVC branches and the graphene, which is influenced by the size and quantity of plasticizer in PVC gel. This innovative approach effectively addresses the limitations posed by graphene's lack of stretchability and the PVC gel's inherently low GF. Additionally, our strain sensor excelled in practical applications, accurately detecting realistic bending motions in human fingers, elbows, and knees, while providing precise angle monitoring within higher strain ranges. Furthermore, our work extends to the development of a PVC‐based posture correction system for band workouts. This multifaceted innovation not only demonstrates potential across various domains but also holds promise in realms such as human‐robot interfaces and wearable electronics, where advanced strain‐sensing capabilities are increasingly valuable.

## 4 Experimental Section

### Synthesis of PVC Gel

The PVC gel solution was prepared using commercial PVC powder (Mw 275000; Scientific Polymer Products, Inc., USA), with tetrahydrofuran (THF 99.5%; DAEJUNG, Korea) as the solvent. DMA, DBA, DOA, and DDA (TCI, Japan) were used as plasticizers. The PVC powder was dissolved in THF, and DMA, DBA, DOA, and DDA were added to the solution, followed by stirring for 12 h at 200 rpm. The ratios of PVC to plasticizer were 1:0.5, 1:1, 1:1.5, and 1:2 (w/w).

### Fabrication of Graphene, PVC Gel, and PVC Gel‐Based Strain Sensor

A graphene solution (Source material product consisting of high‐quality single‐crystal graphene nanosheets, 1.34 wt.% in deionized water; Mexplorer, Korea) was coated on a glass substrate at 5 mm s^−1^ by achieving specified thicknesses (40, 70, and 100 µm) of the dispersed graphene solution in water using a bar coater (BEVS1818, BEVS, Korea). The solution was annealed at 60 °C for 30 min to create a graphene electrode. The PVC solution alone was coated onto the graphene electrodes using a spin coater. Subsequently, 50 mL of PVC gel solution was cast onto the graphene/glass substrate, and the THF was completely evaporated over ≈30 h. Finally, the graphene PVC gel and PVC gel‐based strain sensor were prepared by separating from the glass substrate.

### Material Characterization and Device Performance

The Ultra‐High Resolution Scanning Electron Microscope (SU8020; Hitachi, Tokyo, Japan) was used to characterize the thickness of the graphene and PVC gel layers. The chemical structures of the specimens were analyzed using a Fourier transform infrared (FT‐IR) spectrometer (Spectrometer 100; PerkinElmer, USA). Raman spectra were recorded via a Raman spectrometer (Renishaw inVia Qontor; Renishaw, England) with laser excitation at 532 nm at a power of 1 mW. Surface topography measurements were performed using atomic force microscopy (AFM, XE‐100; Park Systems, Korea) with a Au/Cr‐coated silicon tip. The mechanical properties of the graphene PVC gel were evaluated using a universal testing machine (UTM, ST‐1000; SALT Co., LTD., Korea) according to ASTM D638 (type V) with a crosshead speed of 50 mm/min at room temperature. Uniaxial tensile tests of the graphene PVC gels were performed using dumbbell‐shaped specimens cut from flat drop‐cast films with a thickness of 0.5 mm. The resistance of the graphene PVC gel and PVC gel strain sensor was measured using a Keithley 2450 source meter (Keithley Instruments, USA). The sample was measured by stretching and releasing it at the speed (0.5, 1, 2, 3 cm s^−1^) using a bending machine (SnM, Korea).

## Conflict of Interest

The authors declare no conflict of interest.

## Supporting information



Supporting Information

Supplemental Movie 1

Supplemental Movie 2

Supplemental Movie 3

## Data Availability

The data that support the findings of this study are available from the corresponding author upon reasonable request.
