# Supplementary material for: Constructing Ion‐Bridging Structure with Controlled Cracks in Plasticized PVC with Graphene for Highly Sensitive Strain Sensor with a Wide Strain Range
Source: Adv Sci (Weinh). 2025 Apr 26;12(21):2415998. doi: 10.1002/advs.202415998 (PMC12140297; doi:10.1002/advs.202415998)
Supplement: Supplementary file 1 — Supporting Information [file ADVS-12-2415998-s004.docx]

Supporting Information

**Constructing Ion-Bridging Structure with Controlled Cracks in Plasticized PVC with Graphene for Highly Sensitive Strain Sensor with a Wide Strain Range**

Hyosik Park, Mingyu Kim, Gerald Selasie Gbadam, Cheoljae Lee, Hyeonseo Joo, Sujeong Gwak, Bo-Yeon Lee, Kyeong Nam Kim*, Ju-Hyuck Lee*

**Note S1. FT-IR measurement of PVC gels**

**Figure S4** shows the Fourier transform infrared spectroscopy (FT-IR) spectra of DMA gels, DBA gels, DOA gels, and DDA gels by different plasticizers ratio. The CH_3_ stretching band of all plasticizers appeared at 2955 cm^-1^, but the CH_2_ stretching band at 2928 cm^-1^ and 2870 cm^-1^ appeared only for other plasticizers except DMA. The C-Cl stretching band of PVC appeared at 618 cm^-1^ and the aliphatic ketone stretching (C=O) band of the all plasticizers appeared at 1730 cm^-1^. When the plasticizer concentration increased, the peak of aliphatic ketone stretching (C=O) band increased. The C=O peak is relatively small at low plasticizer concentrations because most plasticizers interact relatively strongly with PVC. However, the C=O peak is shifted to high plasticizer concentrations due to the excess plasticizer's relatively weak interaction (plasticizer-plasticizer). However, due to DMA's smallest molecular weight, excessive plasticizers exist even at low concentrations, so the C=O peak did not shift.

**Note S2. Electrical resistance changes of PVC gels**

**Figures S6-S9** present the resistance changes in different PVC gels (S6: DMA, S7: DBA, S8: DOA, S9: DDA) with plasticizer ratios ranging from 1:0.5 to 1:2. For each plasticizer and corresponding ratios, resistance change intensifies as the applied stretching strain increases. Remarkably, DMA, the smallest plasticizer, demonstrates the most significant resistance change, reaching a maximum of 2.57 (@100% strain) among various plasticizers. This behavior can be attributed to the exceptional mobility of small-sized plasticizer. Furthermore, the addition of more plasticizer leads to an enhancement in resistance change due to improved ionic conductivity and stretchability. In contrast, a reduced amount of plasticizer results in weaker stretchable properties, making it challenging to obtain reliable measurements within a large strain range. Consequently, a higher GF is observed in PVC-based strains sensors when employing a greater quantity of small-sized plasticizer.

**Note S3. Optimization of ion-bridging and fragment/crack size for high gauge factor and mechanical stability**

Regarding the role of the ion bridging, it is important to note that excessive ionic conductivity under strain is undesirable, as it can lead to a decrease in resistance at high strain levels, ultimately degrading the sensor's performance. Therefore, controlling both crack structure (crack size and fragment size) and the mobility of plasticizers is critical. Larger fragment sizes can alter the connectivity of conductive pathways, affecting how ion bridges form and break under strain. While fragment size is inherently linked to crack number, it also plays a direct role in modulating ion conduction dynamics by influencing how conductive pathways evolve under strain. To achieve optimal performance, we found that tuning the plasticizer concentration and mobility is essential. Specifically, by optimizing the DOA concentration at 1:1.5, we ensured that (i) the crack size and fragment size remained within a suitable range to prevent excessive ion bridging, and (ii) the mobility of plasticizers was controlled so that ion bridging gradually decreased under strain rather than collapsing abruptly. This balance between crack structure and ion transport is crucial for achieving high GF while maintaining mechanical stability.


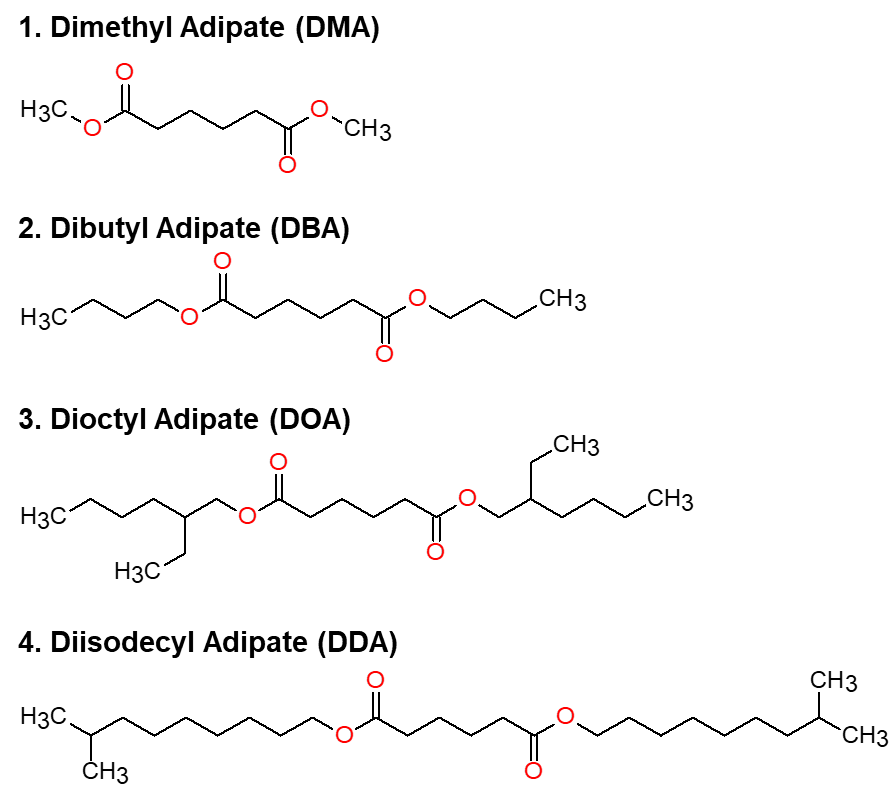


**Figure S1**. The molecular structures of four distinct plasticizers: 1. Dimethyl adipate (DMA), 2. Dibutyl adipate (DBA), 3. Dioctyl adipate (DOA), and 4. Diisodecyl adipate (DDA).


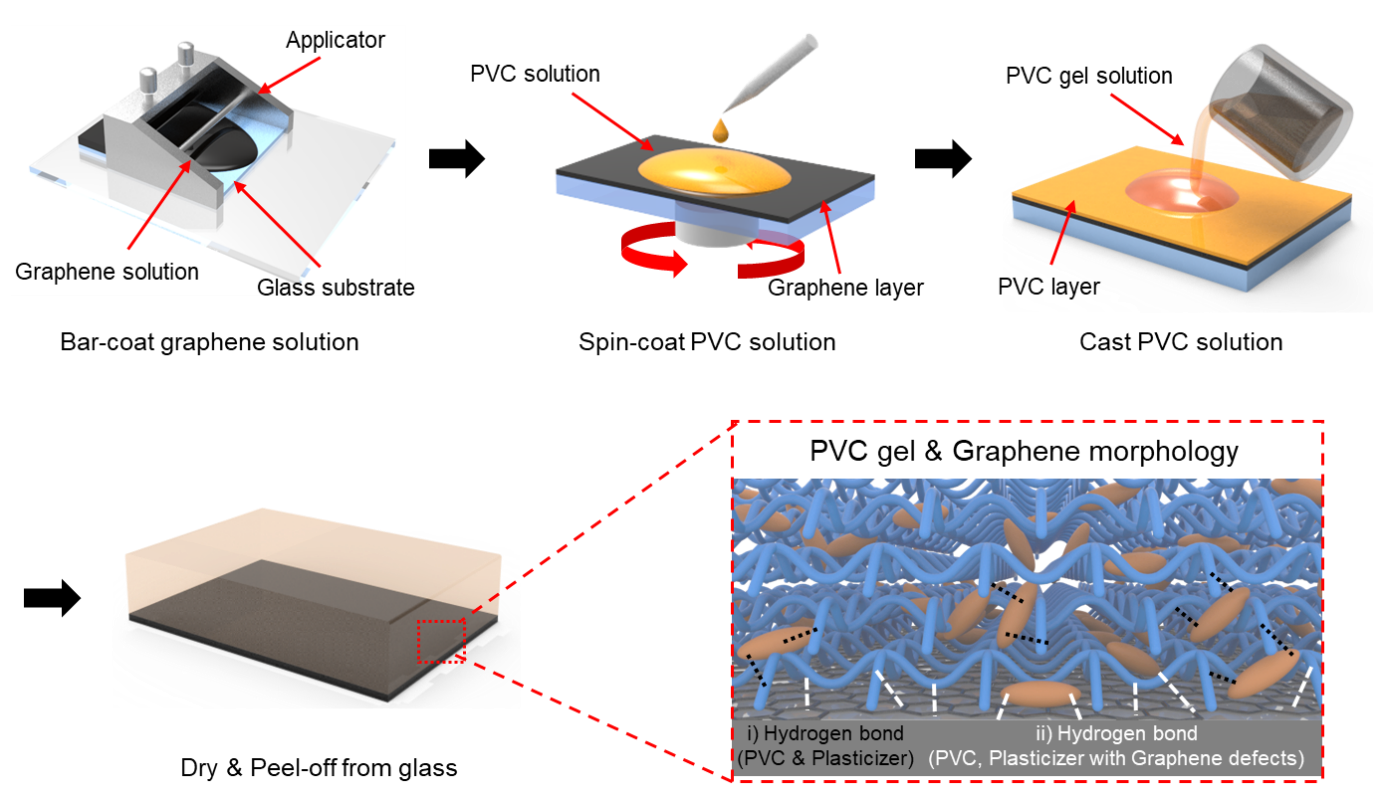


**Figure S2.** Schematic procedures for fabricating a PVC gel/graphene-based strain sensor, highlighting two significant interactions (i: hydrogen bond (PVC & Plasticizer), ii: hydrogen bond (PVC, Plasticizer & graphene defects)) within the PVC gel and the graphene system.


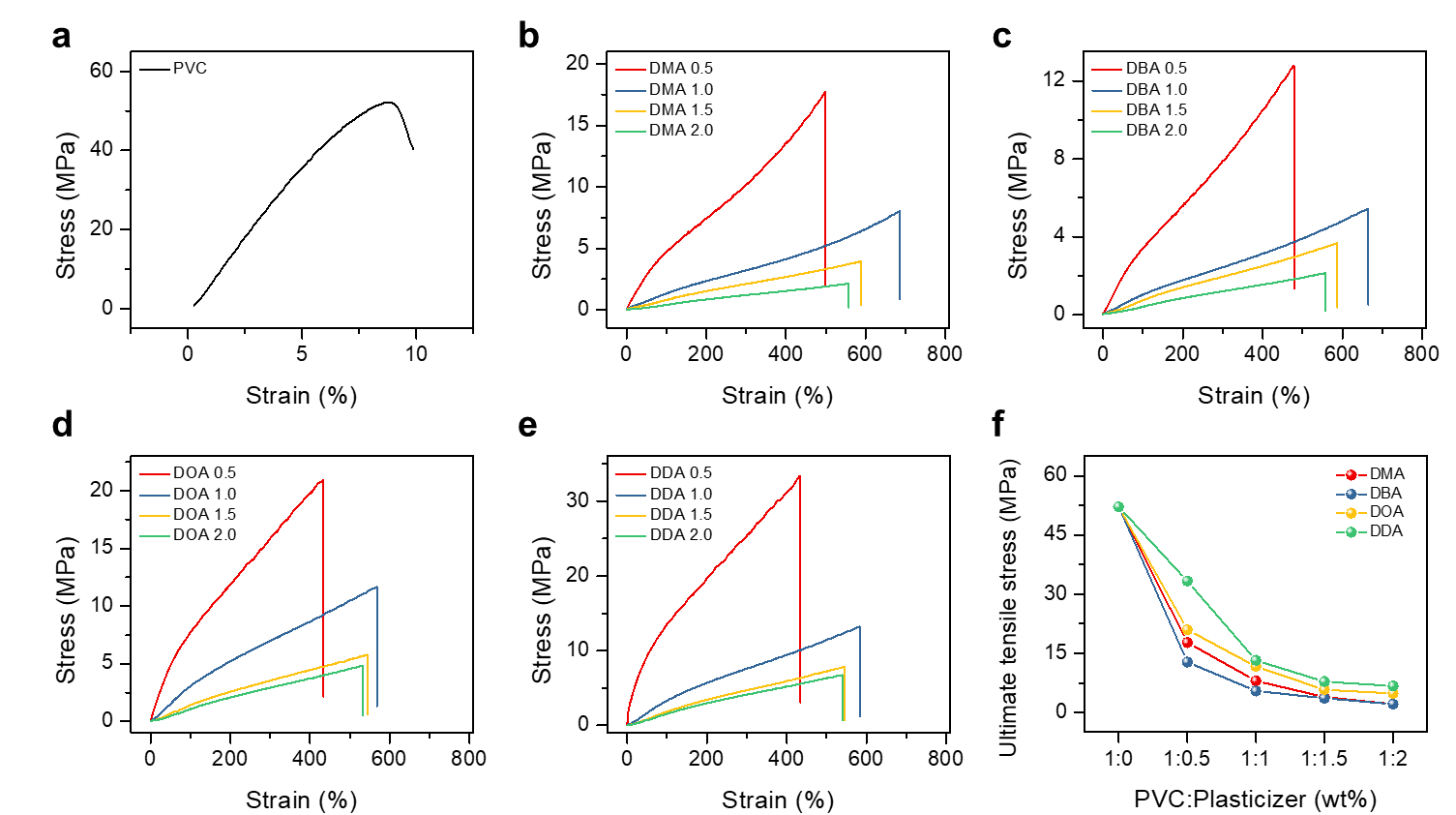


**Figure S3.** Tensile stress-strain curves of PVC and various PVC gels with differing mixing ratios of plasticizers. (a) PVC alone, PVC gels including (b) DMA, (c) DBA, (d) DOA, and (e) DDA. (f) Ultimate tensile stress of PVC and PVC gels.


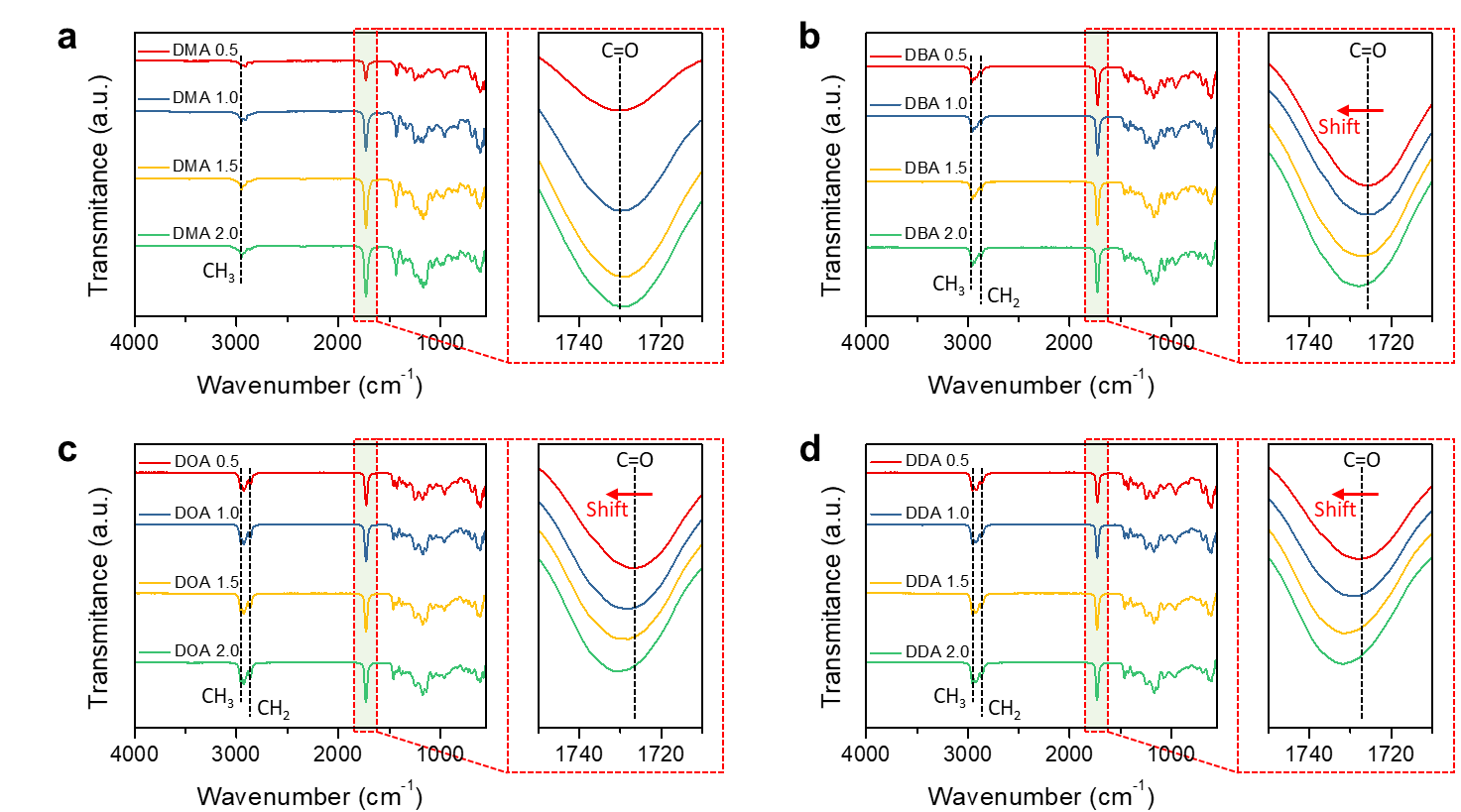


**Figure S4.** The FT-IR spectra of various PVC gels with varying mixing ratios of plasticizers. (a) DMA, (b) DBA, (c) DOA, and (d) DDA.


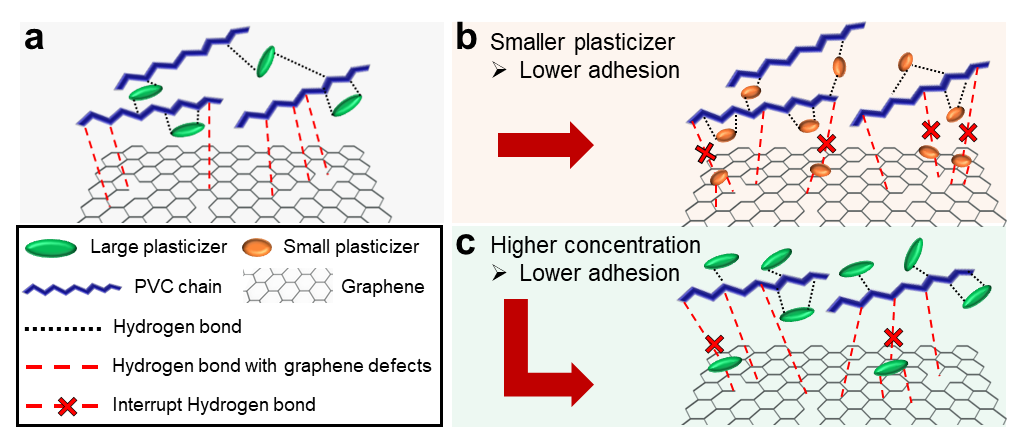


**Figure S5**. Schematic representations illustrate the adhesion capability between PVC gel and graphene based on different plasticizer size and ratios. (a) Low concentration of large plasticizer, (b) low concentration of smaller plasticizer, (c) higher concentration of large plasticizer.


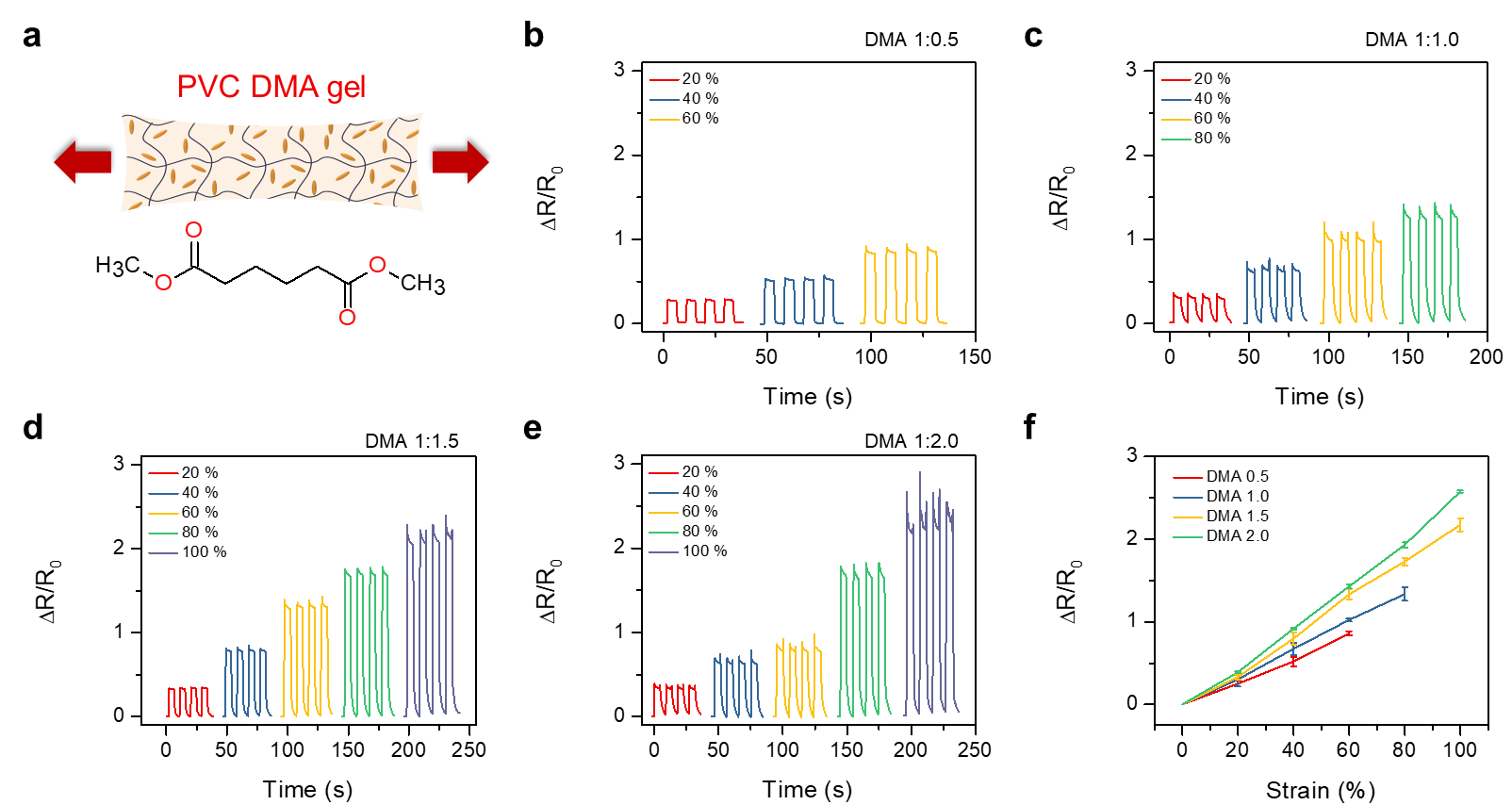


**Figure S6.** (a) Schematic depiction of DMA-based PVC gel. (b-e) Resistance changes of DMA-based PVC gel with differing mixing ratios (0.5, 1.0, 1.5, 2.0) of plasticizers under strain. (f) Resistance profiles for DMA-based PVC gel with varying mixing ratios of DMA under different strains.


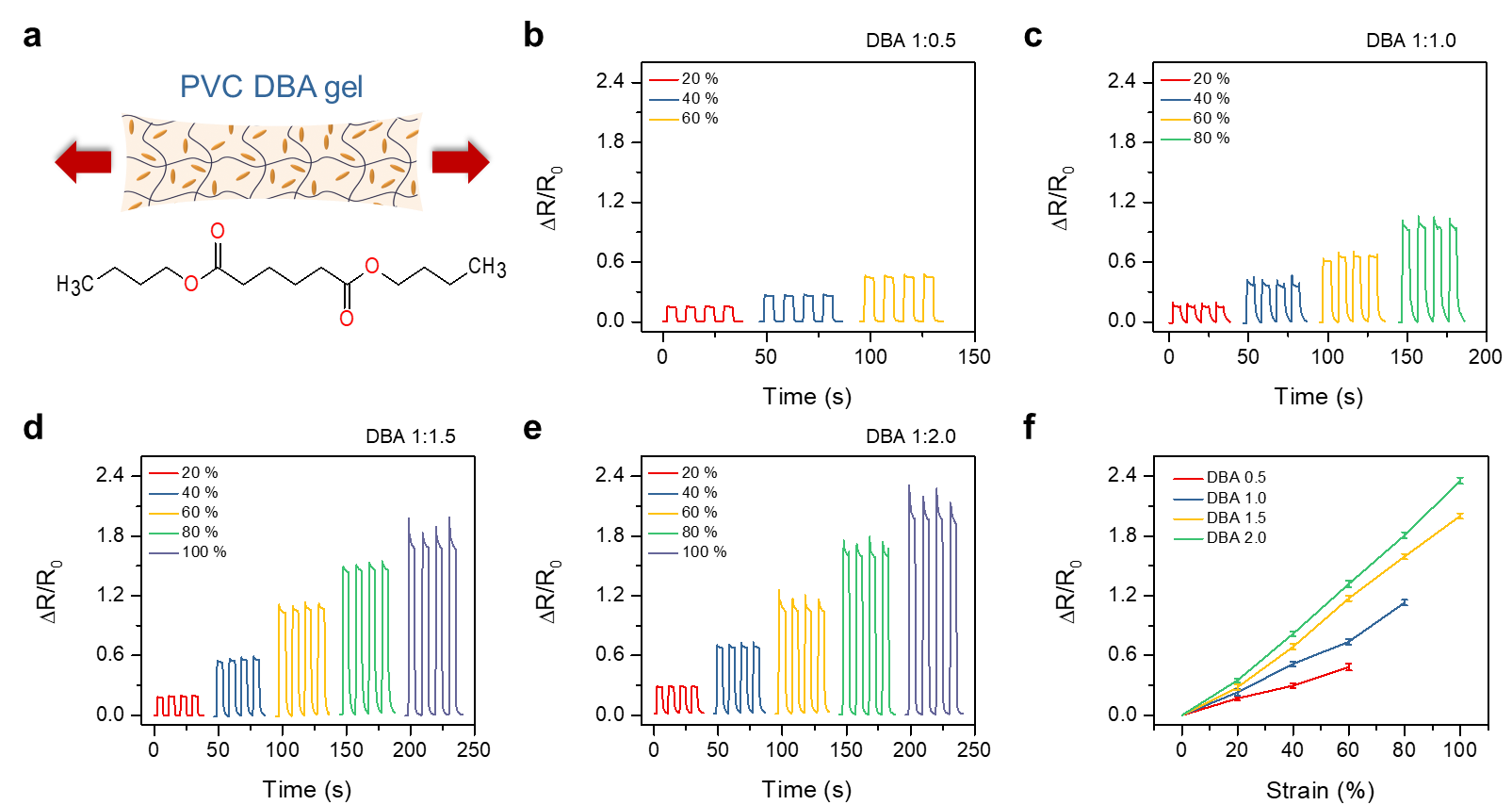


**Figure S7.** (a) Schematic depiction of DBA-based PVC gel. (b-e) Resistance changes of DBA-based PVC gel with differing mixing ratios (0.5, 1.0, 1.5, 2.0) of plasticizers under strain. (f) Resistance profiles for DBA-based PVC gel with varying mixing ratios of DBA under different strains.


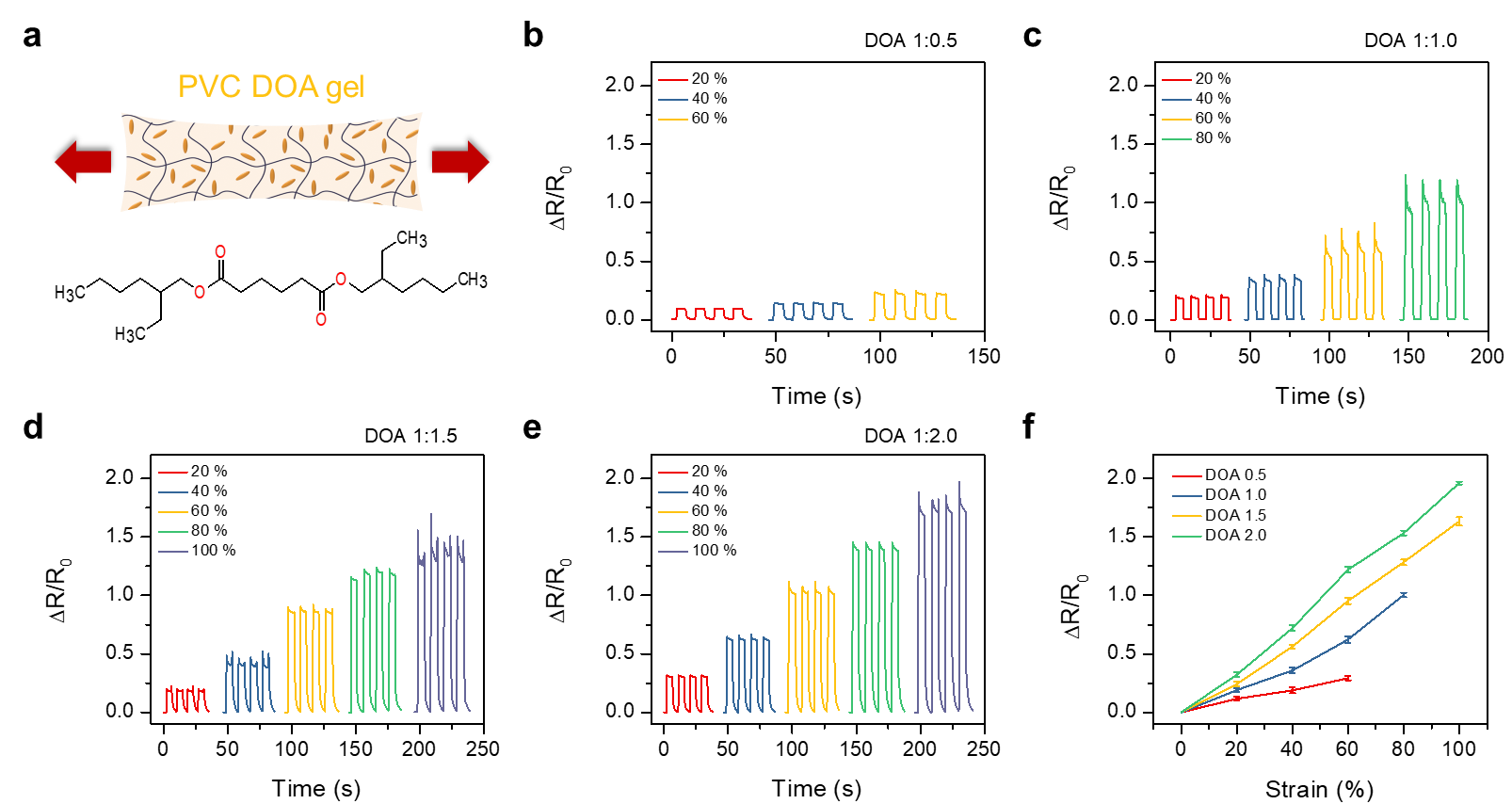


**Figure S8.** (a) Schematic depiction of DOA-based PVC gel. (b-e) Resistance changes of DOA-based PVC gel with differing mixing ratios (0.5, 1.0, 1.5, 2.0) of plasticizers under strain. (f) Resistance profiles for DOA-based PVC gel with varying mixing ratios of DOA under different strains.


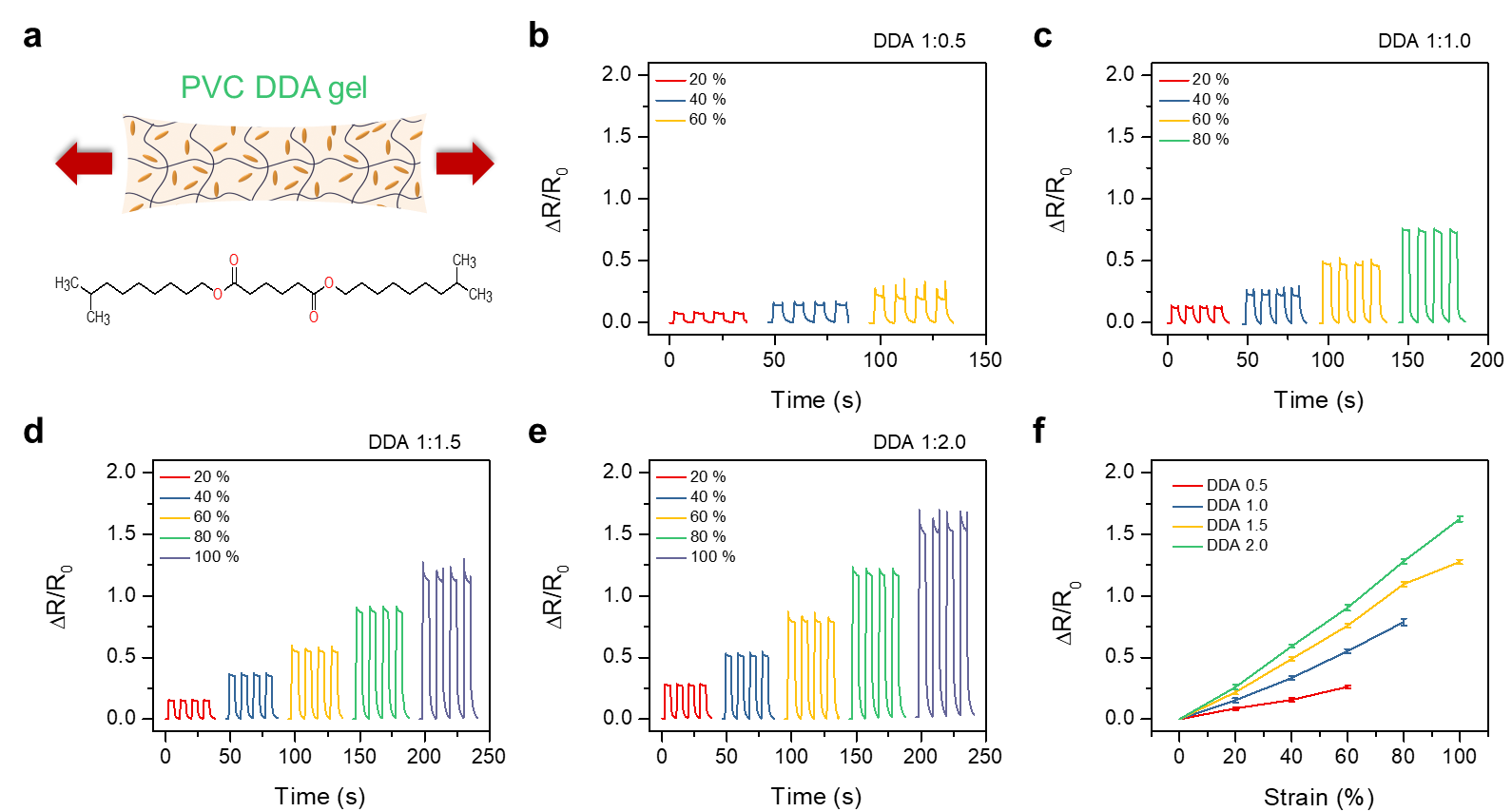


**Figure S9.** (a) Schematic depiction of DDA-based PVC gel. (b-e) Resistance changes of DDA-based PVC gel with differing mixing ratios (0.5, 1.0, 1.5, 2.0) of plasticizers under strain. (f) Resistance profiles for DDA-based PVC gel with varying mixing ratios of DDA under different strains.


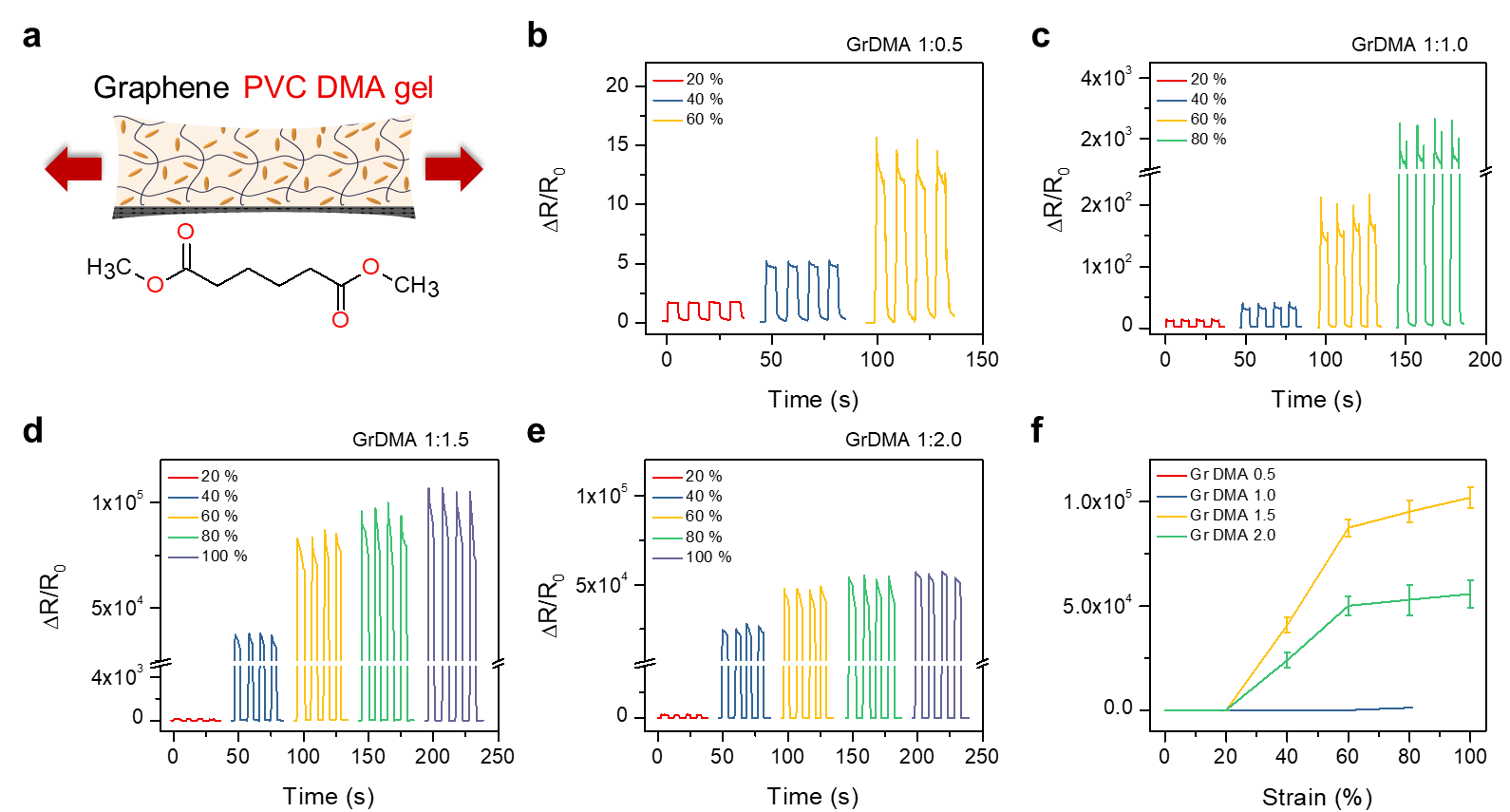


**Figure S10.** (a) Schematic depiction of DMA-based PVC gel/graphene. (b-e) Resistance changes of DMA-based PVC gel/graphene with differing mixing ratios (0.5, 1.0, 1.5, 2.0) of plasticizers under strain. (f) Resistance profiles for DMA-based PVC gel/graphene with varying mixing ratios of DMA under different strains.


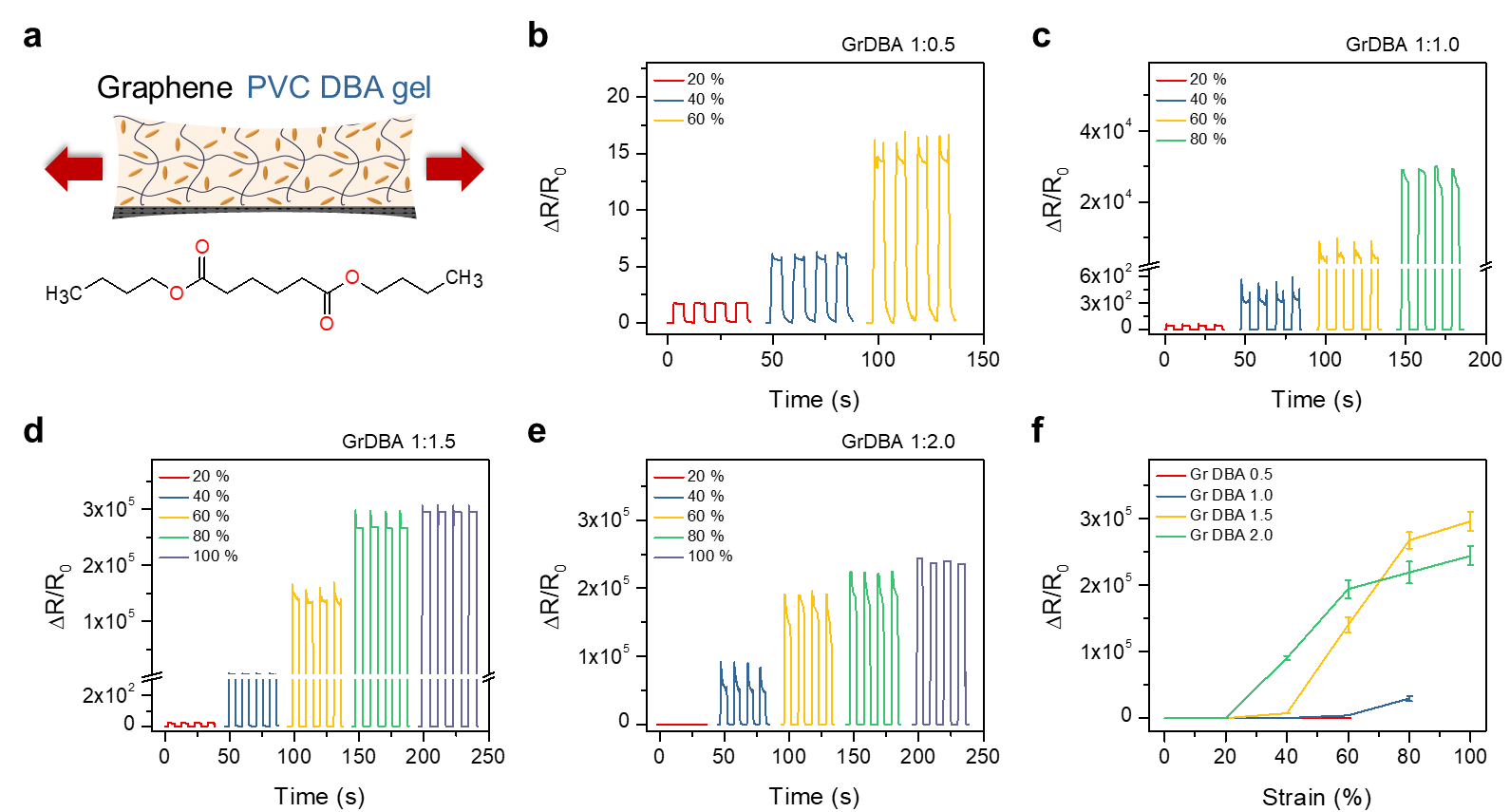


**Figure S11.** (a) Schematic depiction of DBA-based PVC gel/graphene. (b-e) Resistance changes of DBA-based PVC gel/graphene with differing mixing ratios (0.5, 1.0, 1.5, 2.0) of plasticizers under strain. (f) Resistance profiles for DBA-based PVC gel/graphene with varying mixing ratios of DBA under different strains.


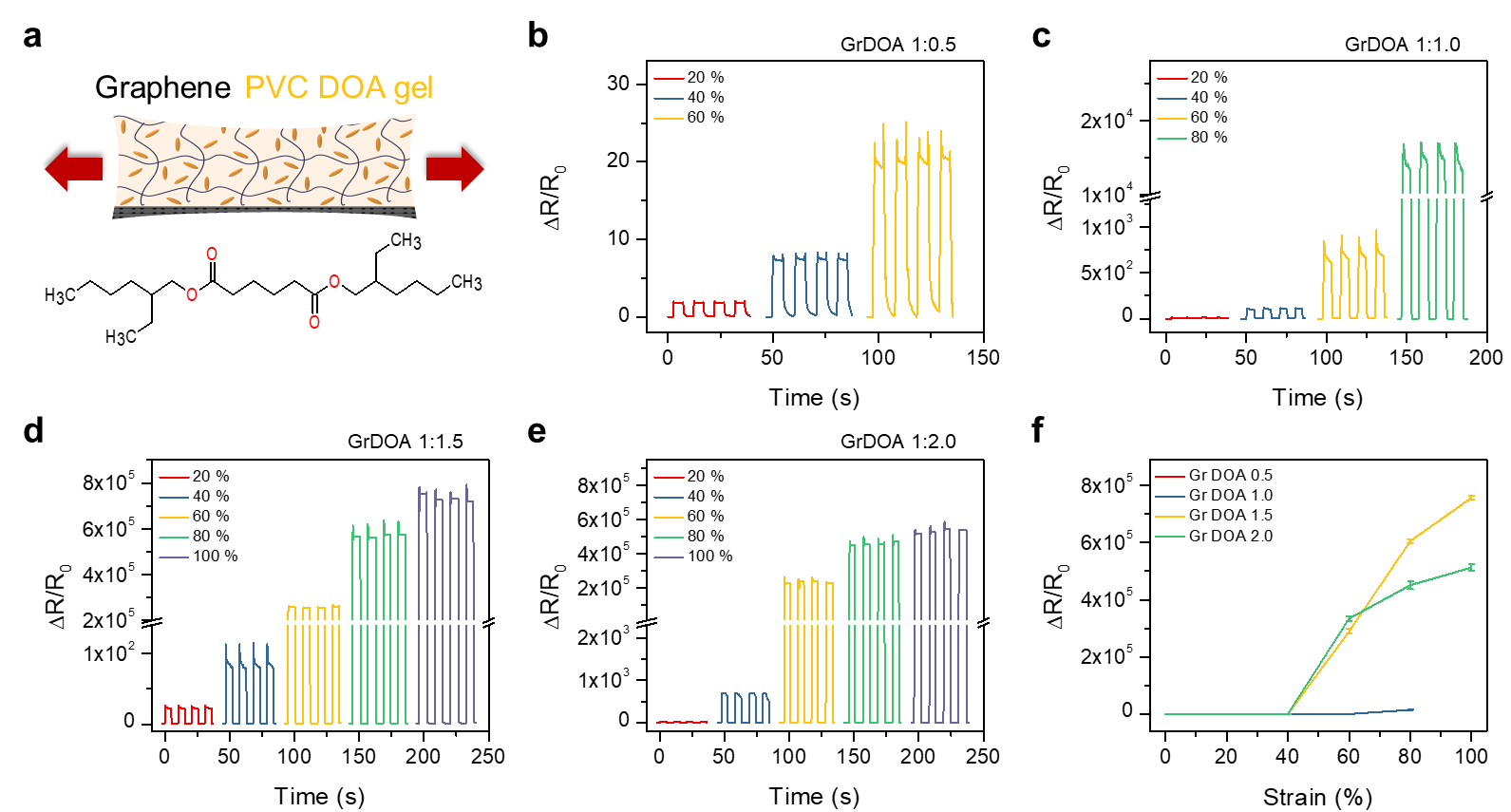


**Figure S12.** (a) Schematic depiction of DOA-based PVC gel/graphene. (b-e) Resistance changes of DOA-based PVC gel/graphene with differing mixing ratios (0.5, 1.0, 1.5, 2.0) of plasticizers under strain. (f) Resistance profiles for DOA-based PVC gel/graphene with varying mixing ratios of DOA under different strains.


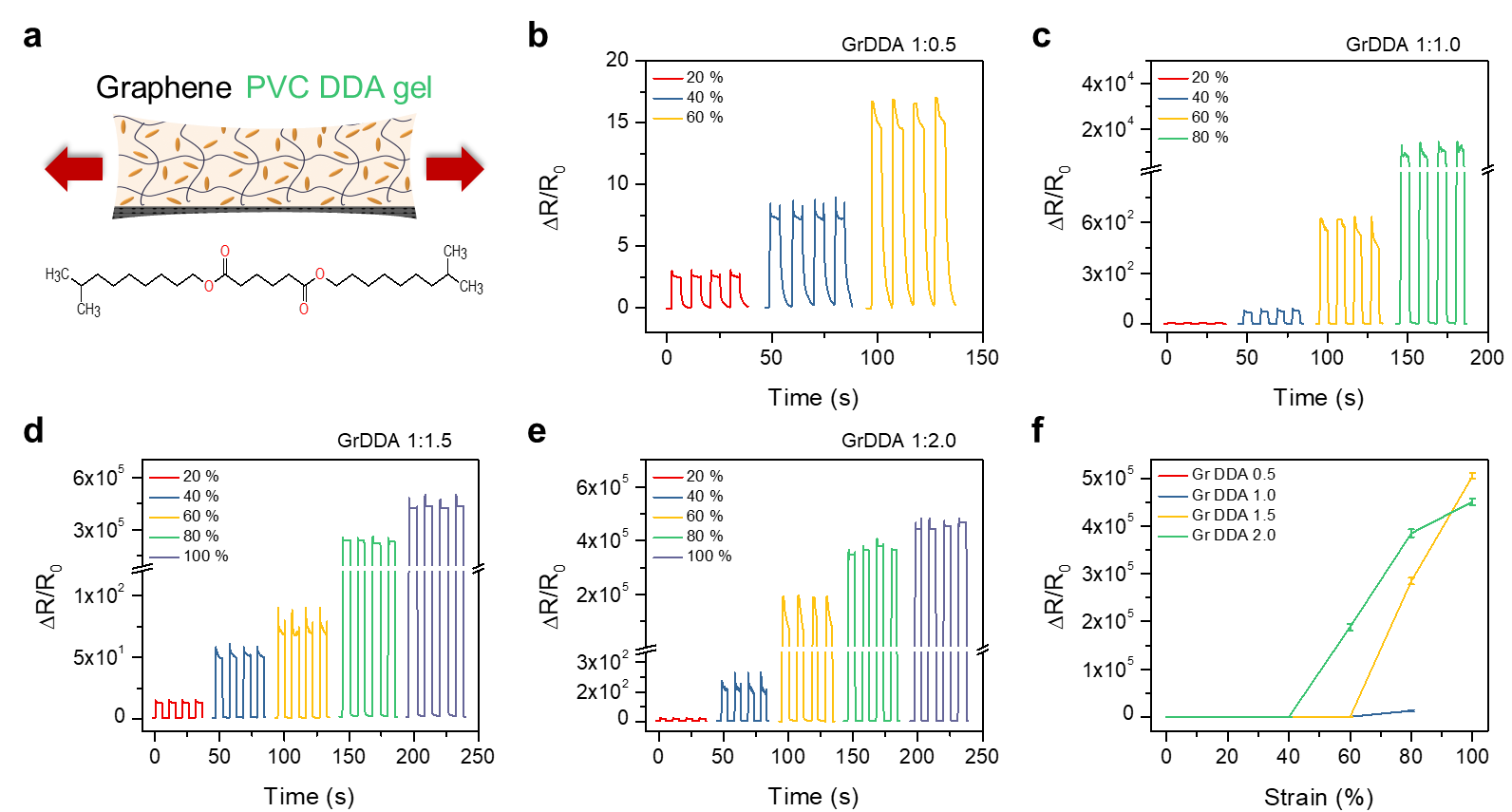


**Figure S13.** (a) Schematic depiction of DDA-based PVC gel/graphene. (b-e) Resistance changes of DDA-based PVC gel/graphene with differing mixing ratios (0.5, 1.0, 1.5, 2.0) of plasticizers under strain. (f) Resistance profiles for DDA-based PVC gel/graphene with varying mixing ratios of DDA under different strains.


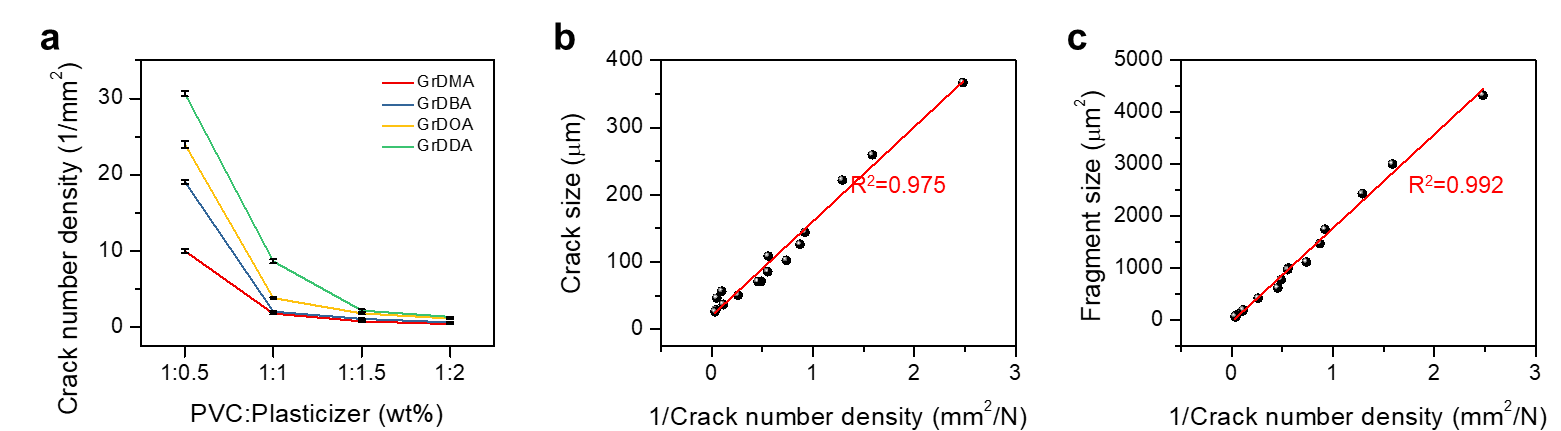


**Figure S14.** (a) Crack number density with different ratios of various plasticizers. b-c. Relationships between crack number density and both crack (b) and fragment sizes (c).

*
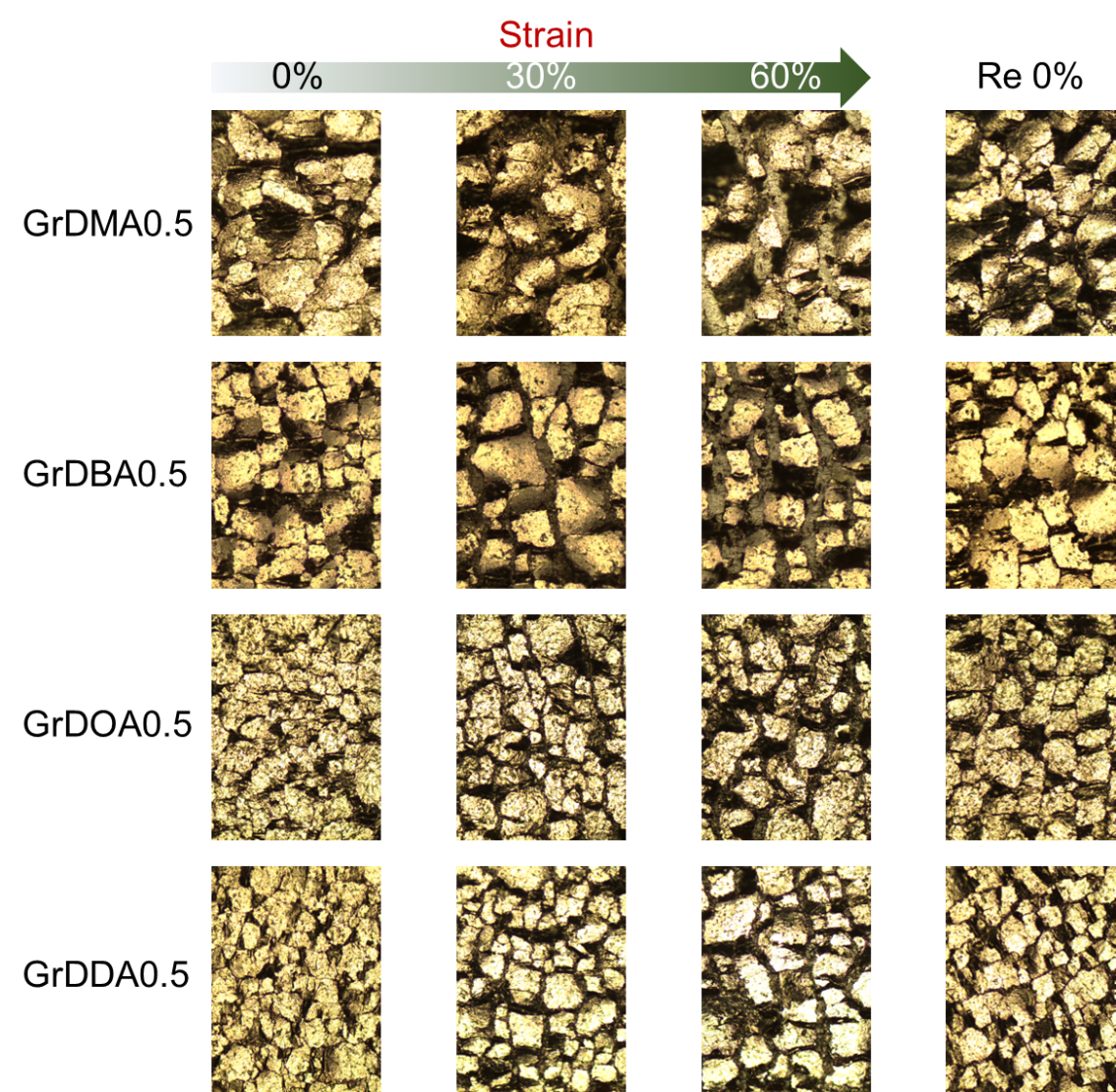
*

**Figure S15**. Optical microscopy images depicting graphene films under various graphene PVC gels with the mixing ratio of 1:0.5 subjected to strains of 0%, 30%, 60% followed by relaxation to 0% (Re 0%).


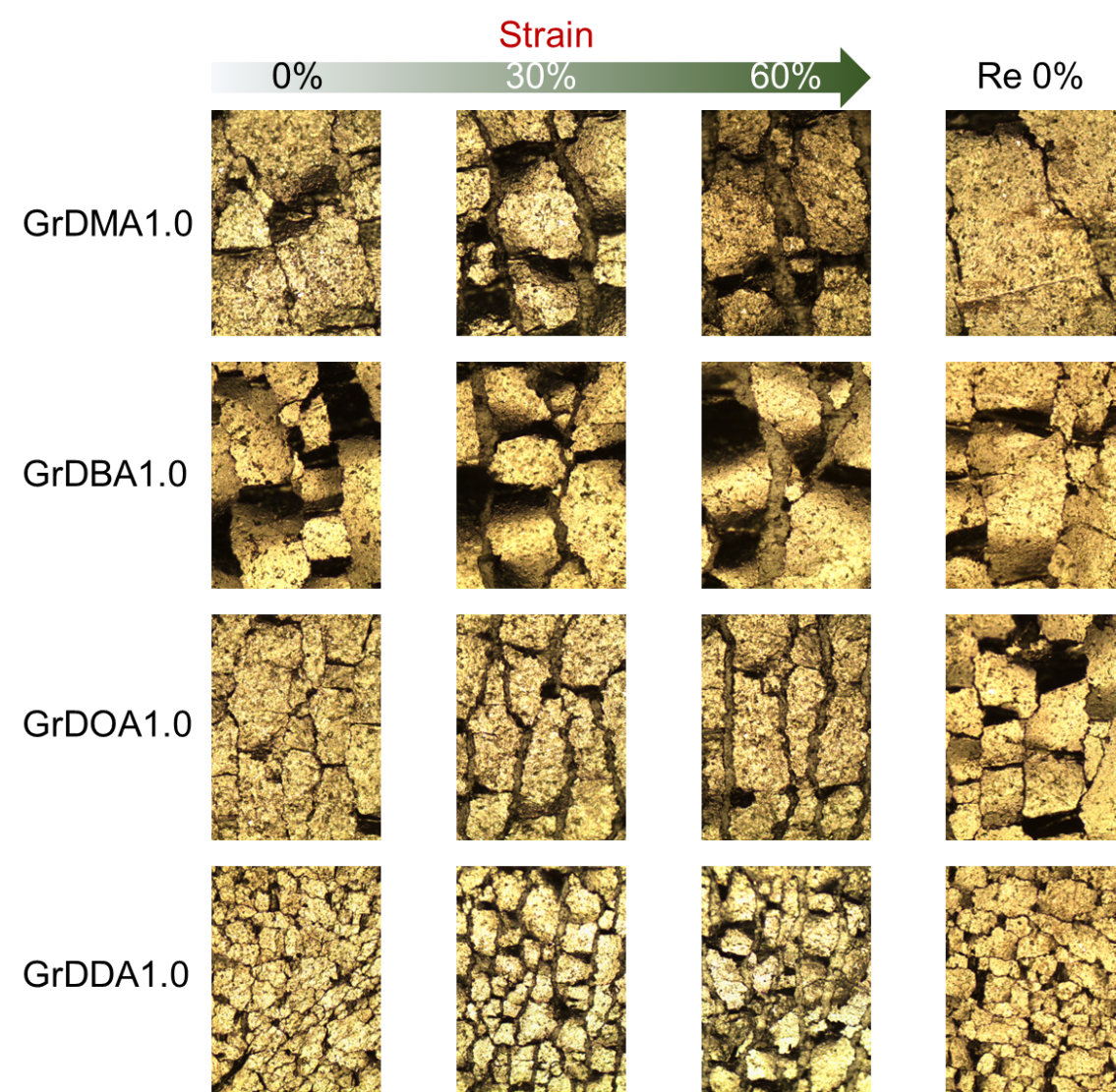


**Figure S16**. Optical microscopy images depicting graphene films under various graphene PVC gels with the mixing ratio of 1:1.0 subjected to strains of 0%, 30%, 60% followed by relaxation to 0% (Re 0%).


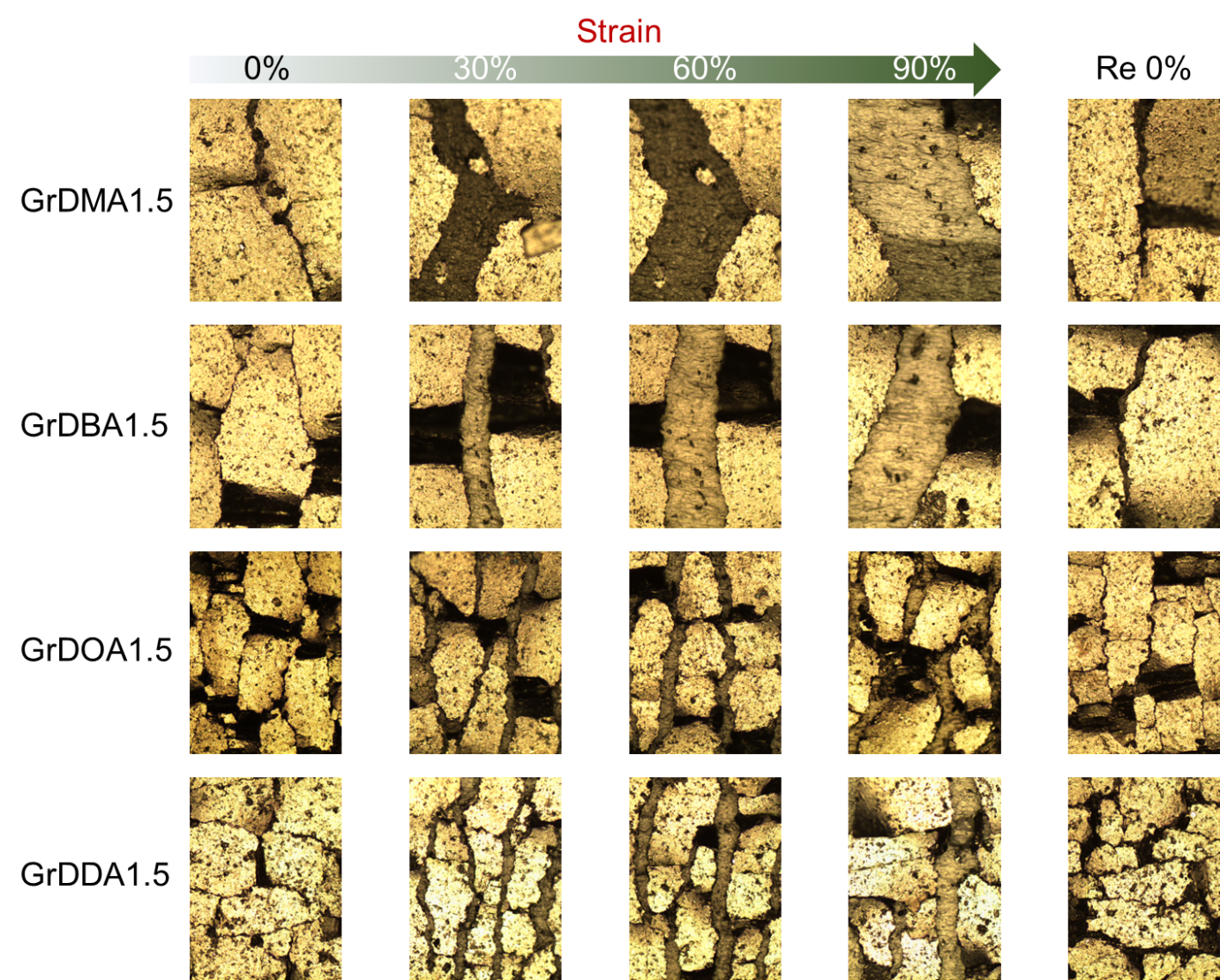


**Figure S17**. Optical microscopy images depicting graphene films under various graphene PVC gels with the mixing ratio of 1:1.5 subjected to strains of 0%, 30%, 60%, and 90% followed by relaxation to 0% (Re 0%).


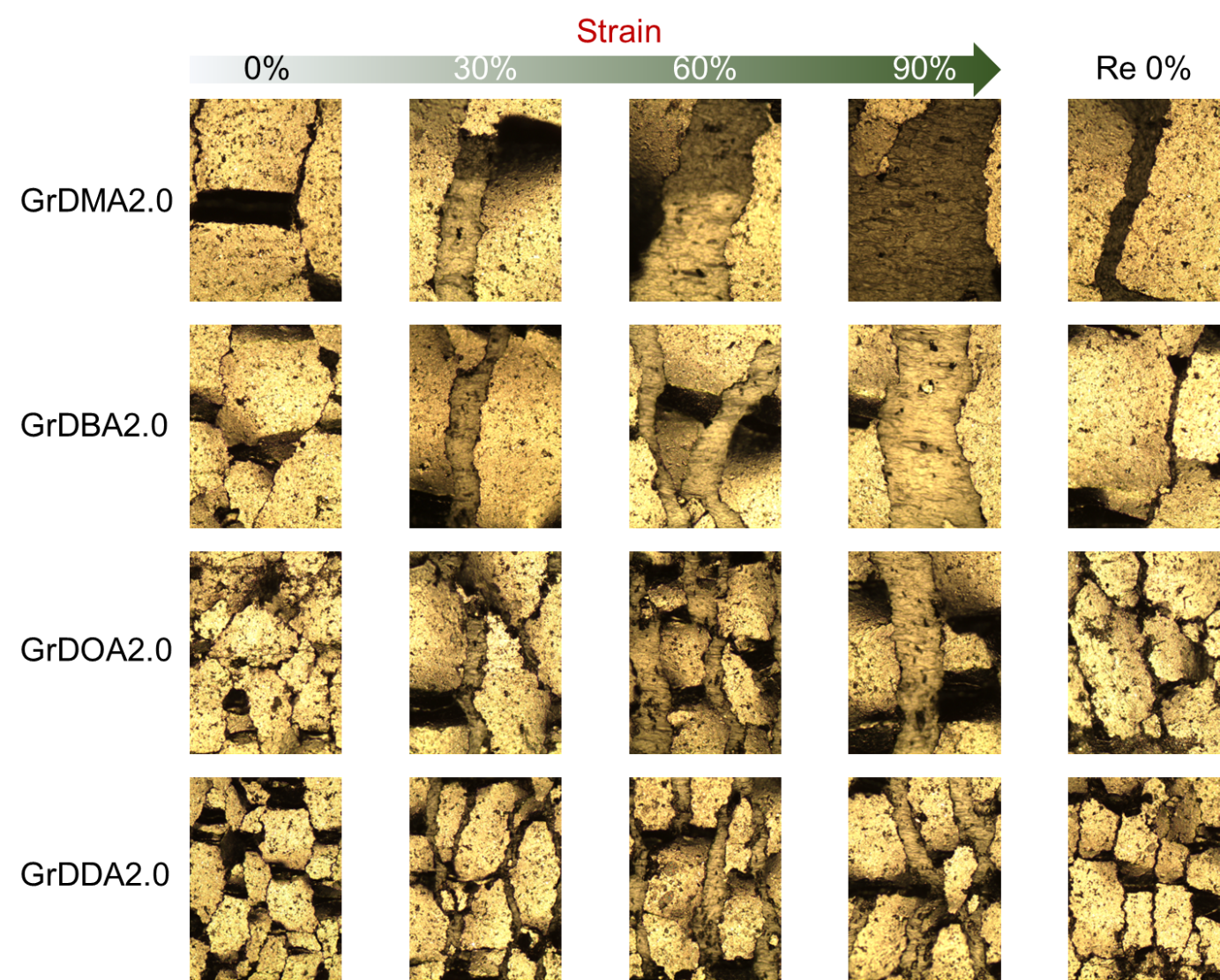


**Figure S18**. Optical microscopy images depicting graphene films under various graphene PVC gels with the mixing ratio of 1:2.0 subjected to strains of 0%, 30%, 60%, and 90% followed by relaxation to 0% (Re 0%).


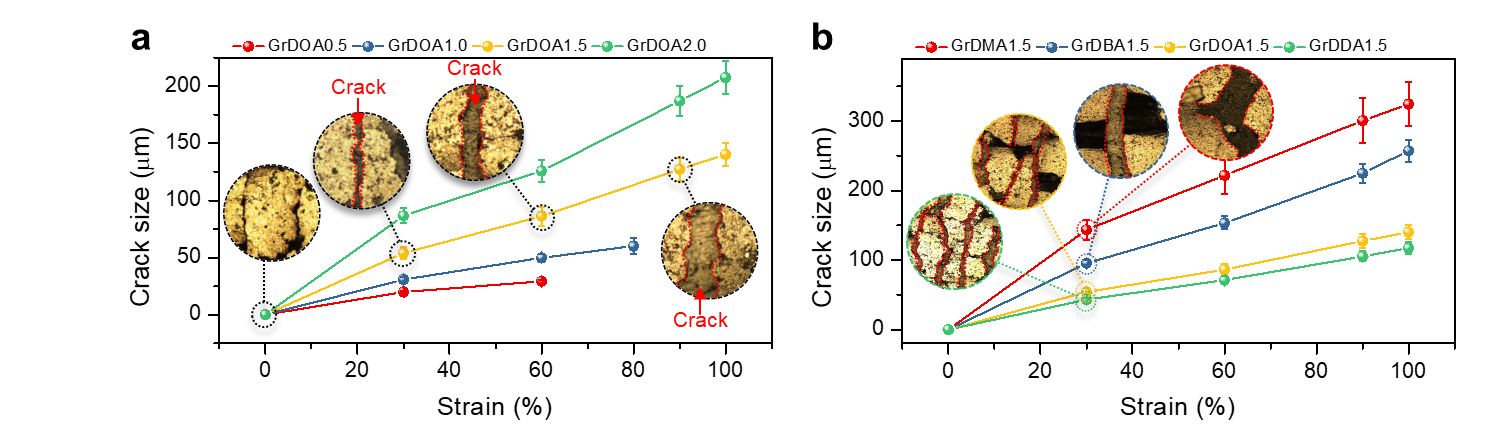


**Figure S19**. a-b. Gap distance between adjacent graphene fragments under varying strains with different DOA ratios (0.5~2.0) (a), and with different plasticizers (DMA1.5, DBA1.5, DOA1.5, and DDA1.5) (b).


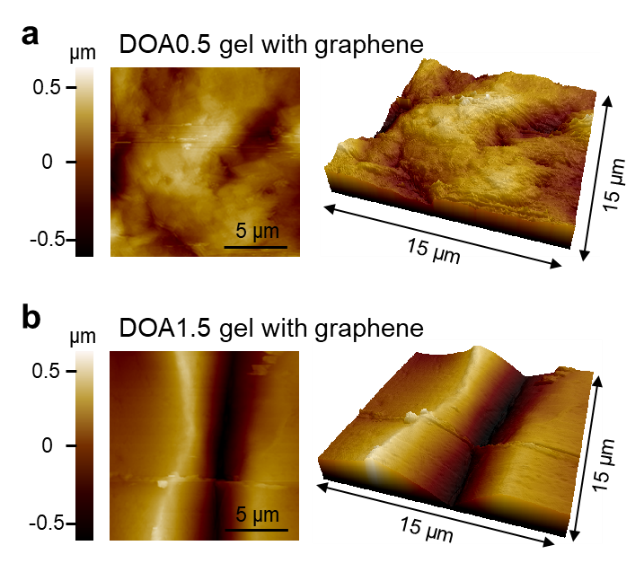


**Figure S20**. a-b. AFM topography image of PVC gels with graphene (a) DOA0.5 and (b) DOA1.5.


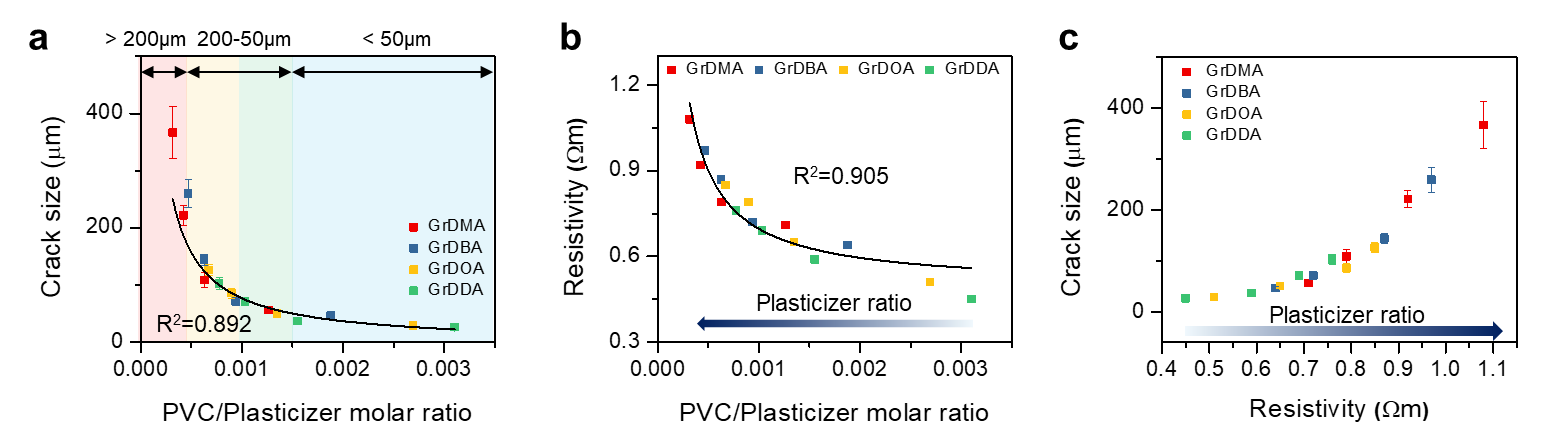


**Figure S21.** Crack modulation based on plasticizer properties. (a) Correlation between PVC/Plasticizer molar ratio and crack size (R^2^=0.892). (b) Correlation between PVC/Plasticizer molar ratio and resistivity of PVC gels with graphene (R^2^=0.905). (c) Correlation between resistivity of PVC gels with graphene and crack size.


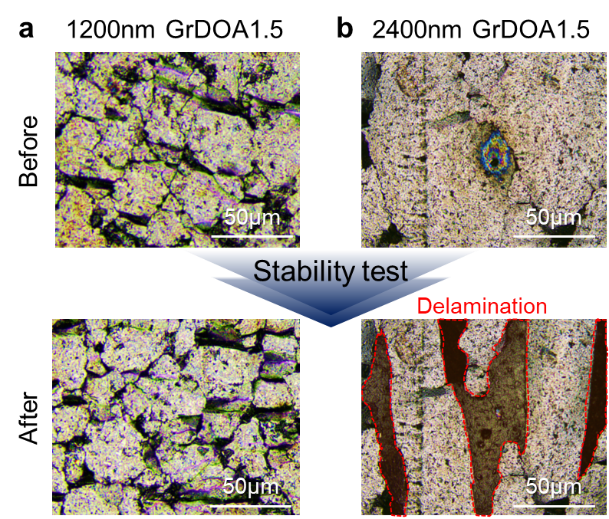


**Figure S22.** Optical microscopy images depicting graphene films under different thickness of graphene before and after stability tests. (a) Stable graphene fragments in the 1,200 nm graphene DOA 1.5 gel. (b) Unstable graphene fragments in the 2,400 nm graphene DOA1.5 gel.


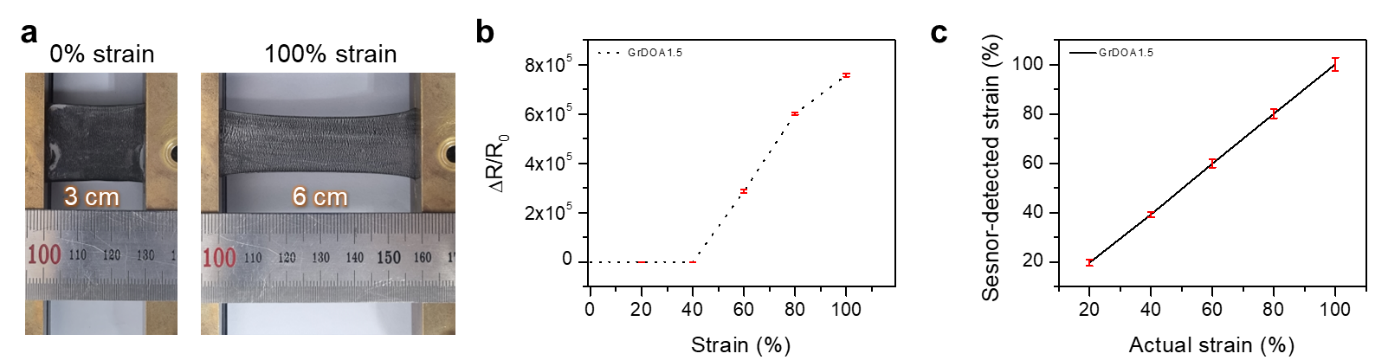


**Figure S23.** The consistency and repeatability tests for 20 fabricated strain sensors based on PVC gel (DOA 1.5) with graphene. (a) Photographic images of PVC gel with graphene with 0 % strain and 100% strain. (b) Resistance changes of 20 fabricated strain sensors. (c) Relationship between actual strain and sensor-detected strain.

**
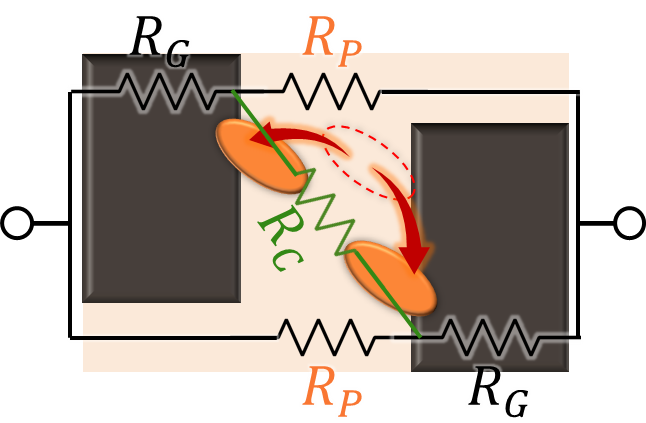
**

**Figure S24**. Circuit diagram of this resistive system based on graphene and PVC gel.


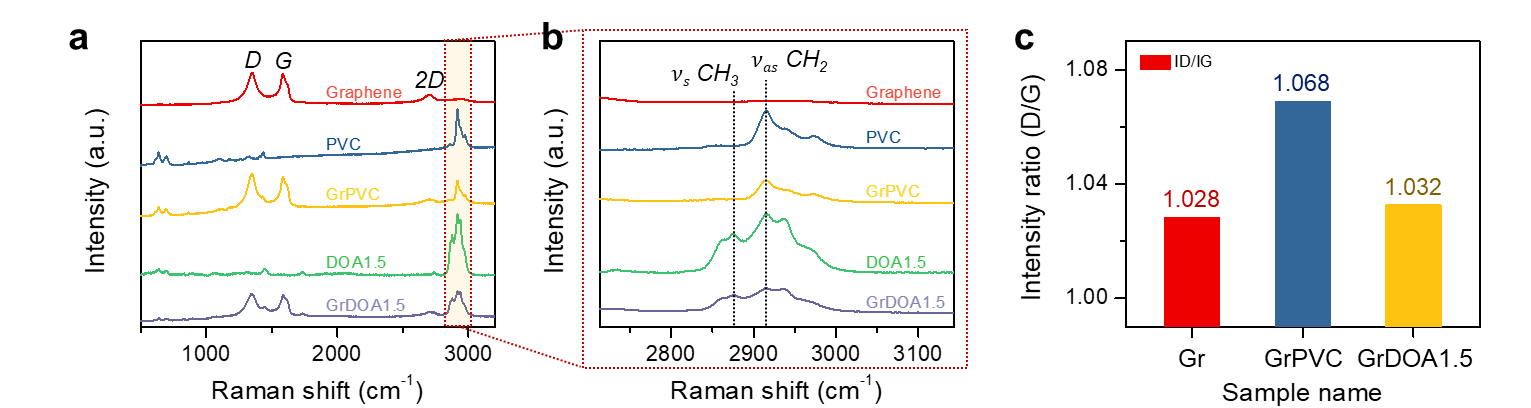


**Figure S25**. (a) Raman spectra of graphene, PVC, Graphene/PVC, PVC gel (DOA 1.5), Graphene/PVC gel (DOA 1.5). (b) Raman spectra of the CH₂ asymmetric stretching (2915 cm^-1^) and the CH₃ symmetric stretching (2870 cm^-1^). (c) Intensity ratio of the D band to the G band in graphene, Graphene/PVC, Graphene/PVC gel (DOA1.5).


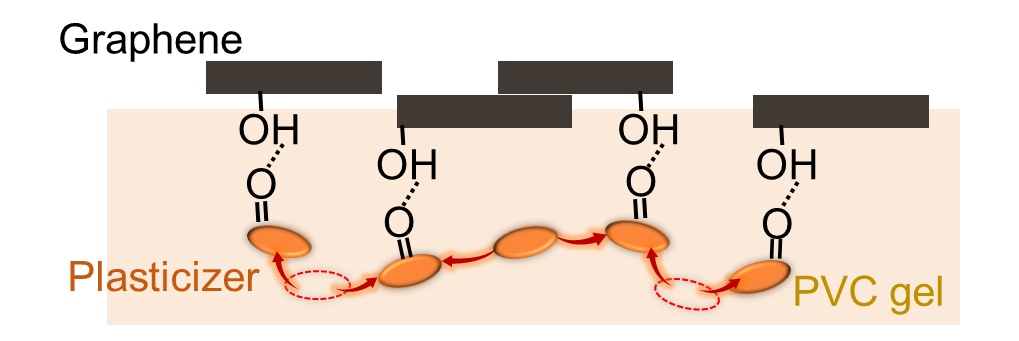


**Figure S26**. Schematic image of ion-bridging effects based on hydrogen bonding interaction between plasticizers and graphene defects.

**Figure S27**. Resistance of PVC gel (DOA 1.5) and PVC gel (DOA 1.5) with graphene measured across the strain range 0% to 100%.


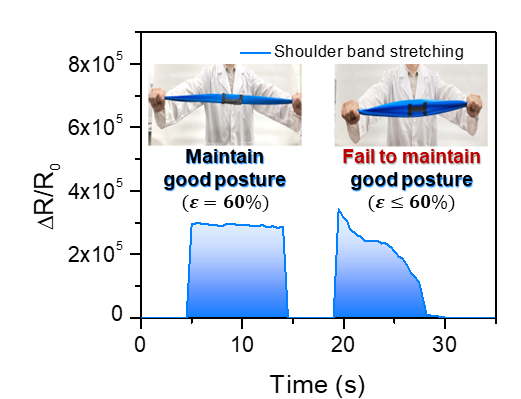


**Figure S28**. Stable resistance change signals during correct posture and unstable signals when posture deviates in strain sensing.


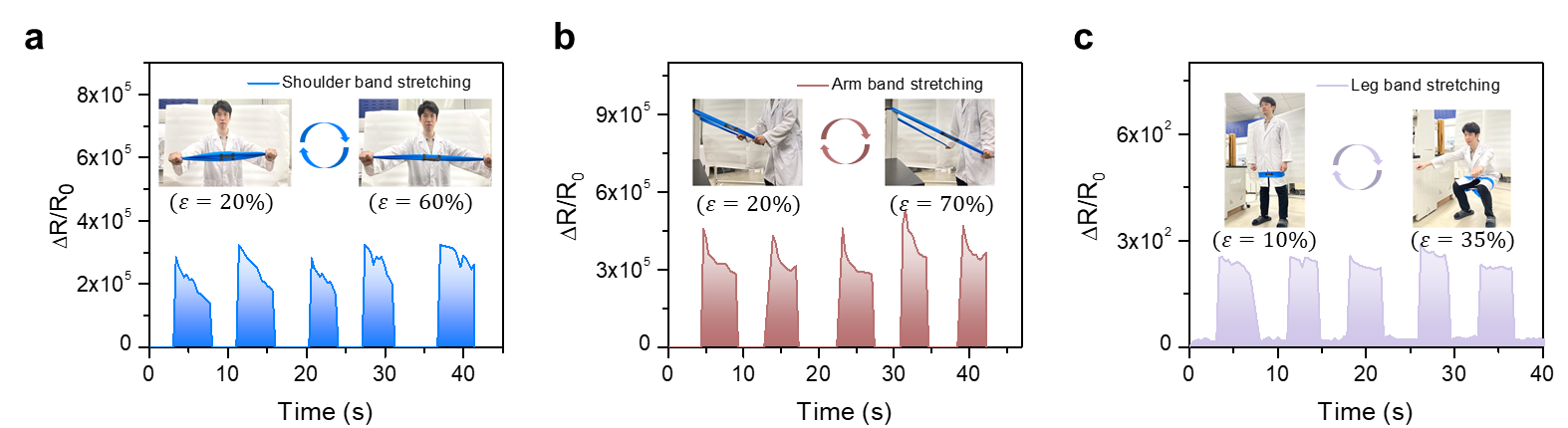


**Figure S29**. Stable posture monitoring during distinct stretching motions, including (a) shoulder band stretching, (b) arm band stretching, and (c) a full squat.

**
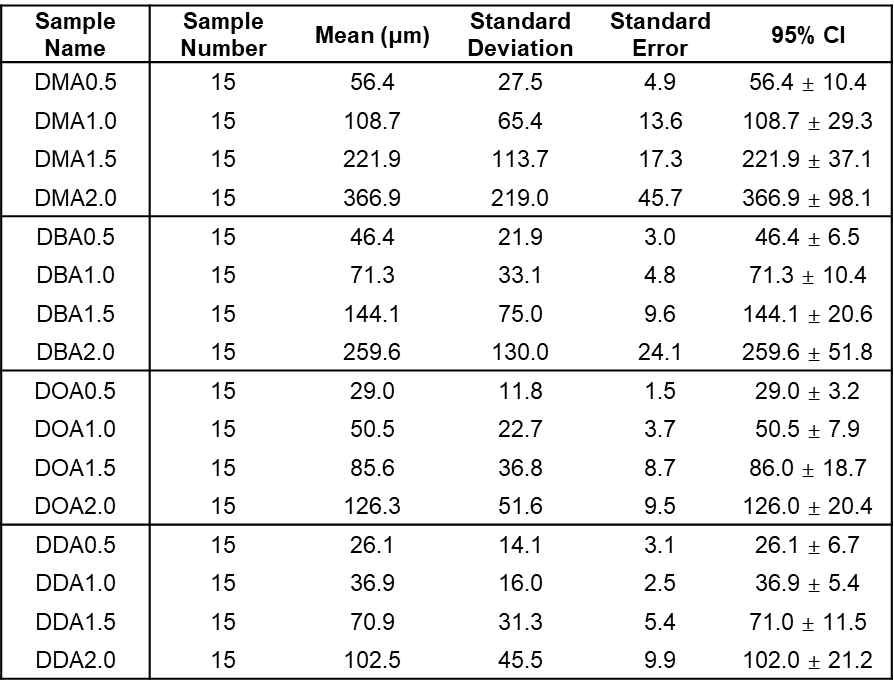
**

**Table S1.** Statistical analysis of crack size under 60% strain as a function of plasticizer concentration and type.

**
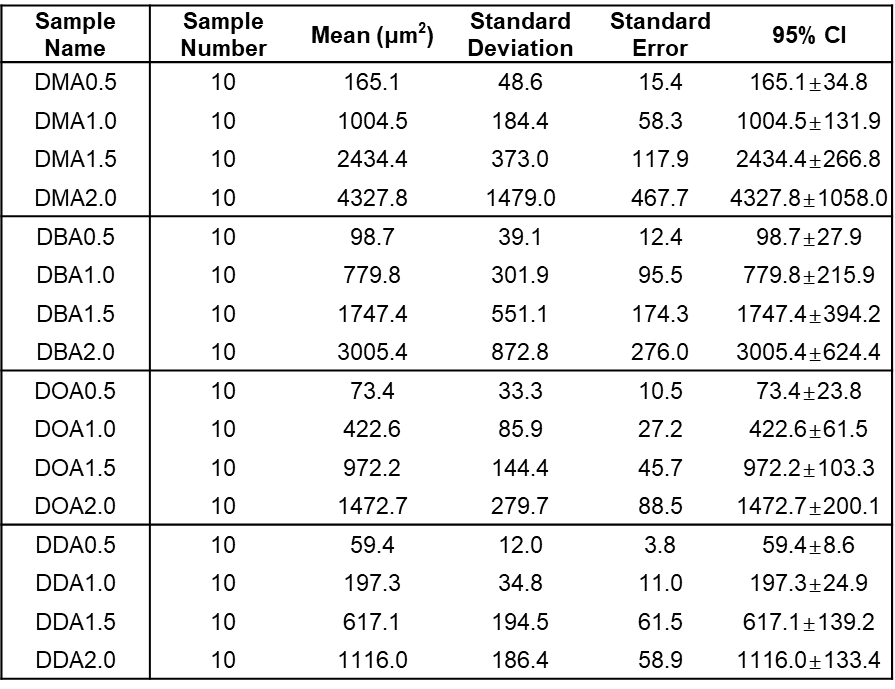
**

**Table S2.** Statistical analysis of fragment size as a function of plasticizer concentration and type.

**
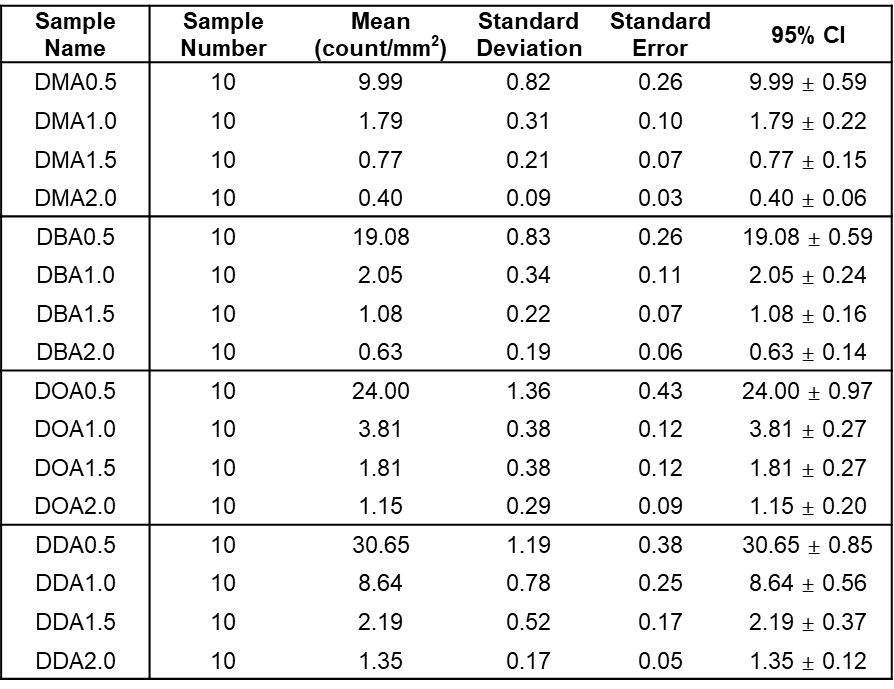
**

**Table S3.** Statistical analysis of crack number density as a function of plasticizer concentration and type.


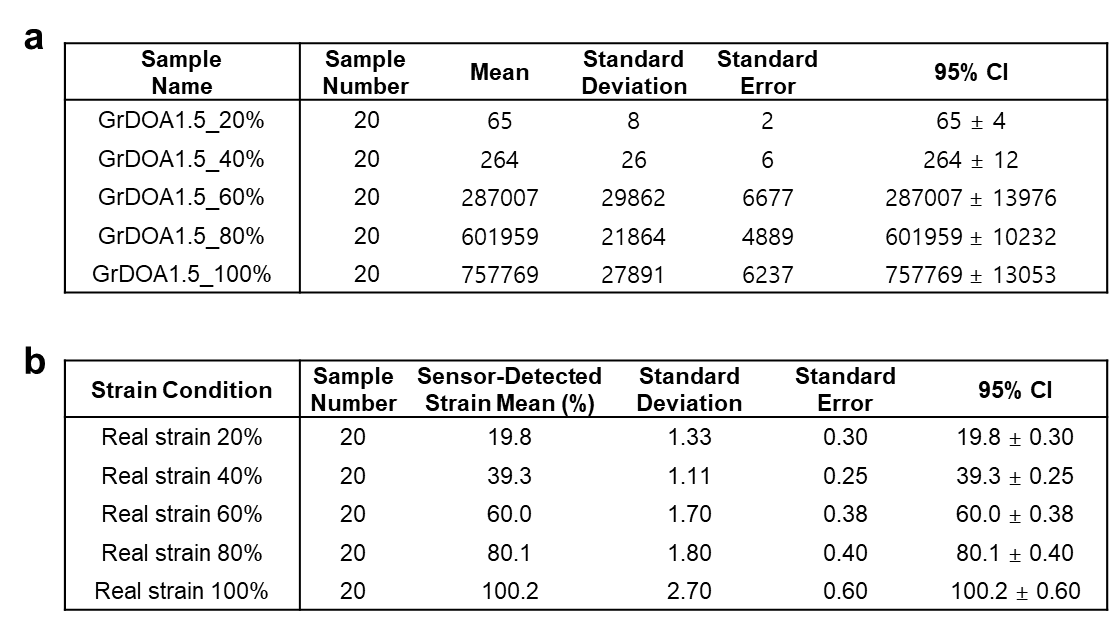


**Table S4.** Statistical summary of the consistency and repeatability tests for 20 fabricated strain sensors based on PVC gel (DOA 1.5) with graphene. (a) Resistance changes at different strains. (b) Relationship between actual strain and sensor-detected strain.
